# Organic Electrochemical Transistors for Neuromorphic Devices and Applications

**DOI:** 10.1002/adma.202515532

**Published:** 2026-01-04

**Authors:** Kexin Xiang, Jiajun Song, Hong Liu, Junxin Chen, Feng Yan

**Affiliations:** ^1^ Department of Applied Physics Research Center for Organic Electronics The Hong Kong Polytechnic University Hung Hom Kowloon Hong Kong 999077 P. R. China; ^2^ Department of Applied Biology and Chemical Technology The Hong Kong Polytechnic University Hung Hom Kowloon Hong Kong 999077 P. R. China; ^3^ Research Institute of Intelligent Wearable Systems The Hong Kong Polytechnic University Hung Hom Kowloon Hong Kong 999077 P. R. China

**Keywords:** artificial neural network, artificial neuron, artificial synapse, mixed ionic–electronic conductor, neuromorphic engineering, organic electrochemical transistor

## Abstract

Neuromorphic engineering, an interdisciplinary field bridging bioelectronics and neuroscience, endeavors to address the bottleneck of the von Neumann architecture by constructing hardware‐level artificial neural networks (ANNs) and replicate the complicated architecture and functionality of the human brain, heralding a new era of intelligent sensing, processing, and computing systems. Organic electrochemical transistors (OECTs), which operate via the bulk doping of organic mixed ionic–electronic conductors, are emerging as promising platforms for neuromorphic devices that emulate neuronal and synaptic activities while seamlessly integrating with biological systems. OECTs offer several advantages, including compatibility with flexible and stretchable substrates, tunable ionic and electronic conductivity, multimodal sensing capability, and operation at low voltages. This review aims to provide a comprehensive and state‐of‐the‐art vista of the rapidly advancing field of OECT‐based neuromorphic devices, including organic electrochemical neurons, organic electrochemical synapses, and their integrated devices. Particular emphasis is placed on their ability to perform neuromorphic functions and diverse applications in neuromorphic computing and flexible biointerfaces. Conclusions, remaining challenges, and future prospects for the development of OECT‐based neuromorphic devices are finally outlined.

## Graphical abstract

Organic electrochemical transistors are emerging as promising platforms for neuromorphic devices that emulate neuronal and synaptic activities and can seamlessly integrate with biological systems. This review focuses on resultant organic artificial neurons, synapses, and integrated devices, with an emphasis on their ability to perform neuromorphic functions and diverse applications in neuromorphic computing and flexible biointerfaces.

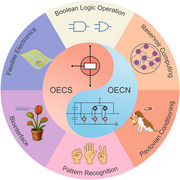

## 1 Introduction

Thanks to the invention of silicon‐based transistors by William Shockley and colleagues in the Bell Laboratory, artificial neural networks (ANNs) and artificial intelligence (AI) based on modern computers have undergone remarkable development over the past few decades. However, modern computers are constructed upon the von Neumann architecture, which faces a significant computational bottleneck due to the growing mismatch in data transfer rates between the central processing unit (CPU) and main memory. To address this constraint, a transition of ANNs from software‐based implementations to hardware‐based architectures is increasingly anticipated. This shift aims to integrate computational units and memory at the physical level, as the von Neumann separation remains fundamentally fixed in hardware. With the introduction of time‐domain processing and asynchronous computation, spiking neural networks (SNNs) offer a promising strategy for enabling neuromorphic hardware systems.^[^ ^1^, ^2^, ^3^ ^]^

Neuromorphic engineering, an interdisciplinary field bridging bioelectronics and neuroscience, endeavors to address the bottleneck of the von Neumann architecture by constructing hardware‐level ANNs to replicate the complicated architecture and functionality of the human brain, heralding a new era of intelligent sensing, processing, and computing systems. The human brain comprises an intricately connected neural network of ≈10^12^ neurons and 10^15^ synapses, operating in parallel to perform complex cognitive functions.^[^ ^4^ ^]^ Considering that each neuron links to thousands of synapses, enabling effective parallel processing, the human brain exhibits remarkable efficiency in solving complex problems, while consuming only ≈20  W of power and operating at a frequency of ≈10  Hz.^[^ ^5^, ^6^, ^7^ ^]^ Neurons and synapses are the fundamental building blocks of neural systems: neurons are responsible for processing and transmitting signals when stimulation intensities exceed a defined threshold, while synapses connect two adjacent neurons to form memory and certain plasticity functionalities. A neuromorphic device refers to electronic hardware that is designed to emulate the structure and functionality of the human brain, enabling intelligent systems capable of rapid and adaptive responses to environmental stimuli. By scaling up hardware components to support massively parallel computing, advanced cognitive functionalities can be achieved at a low energy cost, far beyond the capability of traditional computing devices. In 1990, Mead proposed the first documentation of devices and circuits that possess significant characteristics akin to information‐processing systems in organisms, and put forward the concept of neuromorphic electronic systems.^[^ ^8^ ^]^ To systematically and precisely imitate the operations of the human brain, three aspects of research must be addressed: the development of artificial neurons, artificial synapses, and system‐level architectures that emulate biological neural networks.^[^ ^9^, ^10^ ^]^ Neuromorphic devices offer efficient, fault‐tolerant, and compact means for figuring out intricate practical tasks, highlighting their applications in pattern recognition,^[^ ^11^ ^]^ prosthetics,^[^ ^12^ ^]^ and motion control,^[^ ^13^ ^]^ etc.

Similar to biological neurons, the fundamental feature of artificial neurons is event‐based perception, which maximizes the information communication efficiency and minimizes the power consumption by processing and transmitting signals only when necessary or required. Benefiting from the mature fabrication techniques of silicon‐based integrated circuits, most existing artificial neurons are made of silicon and have been adopted in various perception scenarios, including touch,^[^ ^14^ ^]^ smell,^[^ ^15^ ^]^ and vision.^[^ ^16^, ^17^ ^]^ However, silicon‐based neurons lack biocompatibility and biochemical responsiveness, and they are hindered by their rigidity, fragility, and fabrication complexity. Consequently, a variety of alternative materials have emerged to construct artificial neurons, including 2D chalcogenide,^[^ ^18^, ^19^ ^]^ ferroelectric materials,^[^ ^20^, ^21^ ^]^ phase‐change materials.^[^ ^22^, ^23^ ^]^ Likewise, artificial synapses have been widely realized by silicon‐based transistors in the past decades, offering the basis of memory and learning in ANNs.^[^ ^24^, ^25^ ^]^ Beyond silicon, various materials have been employed in artificial synapses, such as metal oxide,^[^ ^26^, ^27^ ^]^ 2D chalcogenide,^[^ ^28^, ^29^ ^]^ ferroelectric materials,^[^ ^30^, ^31^, ^32^ ^]^ and phase‐change materials.^[^ ^33^, ^34^ ^]^ Nevertheless, the conventional choices of silicon and other inorganic materials as the primary composition of artificial neurons and synapses in neuromorphic systems impose limitations on their applications, particularly in terms of flexibility, biocompatibility, and multimodal sensing capability.

**Figure 1:**
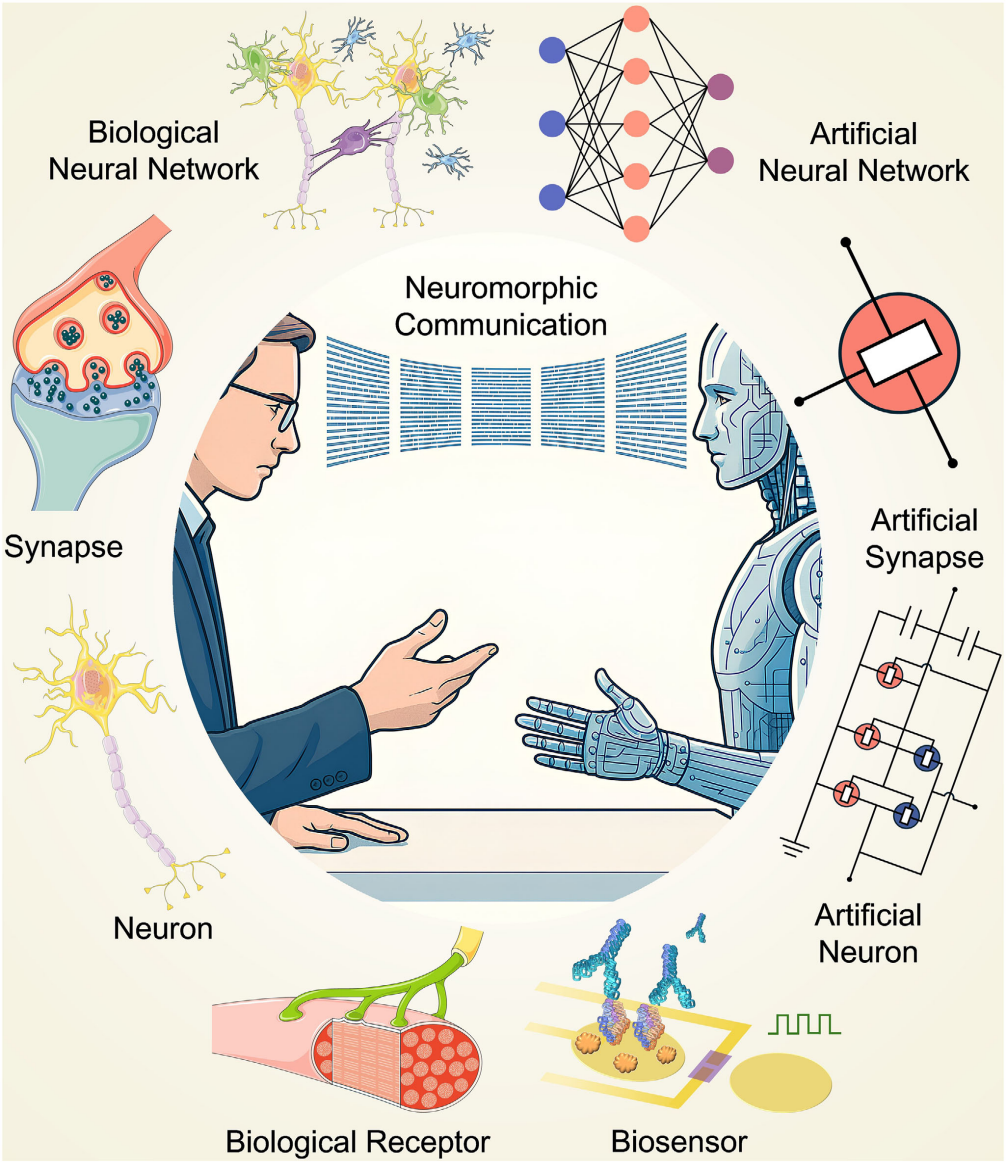
Overview of the OECT‐based neuromorphic devices and their biological counterparts. The graphics of the biosensor are reproduced with permission.^[^ ^82^ ^]^ Copyright 2021, American Association for the Advancement of Science. The graphics of biological receptors, neurons, synapses, and biological neural networks are adapted from Servier Medical Art templates, which are licensed under a Creative Commons Attribution 3.0 Unported License; https://smart.servier.com/.

Notably, organic mixed ionic–electronic conductors (OMIECs) provide a promising paradigm for realizing biorealistic applications of neuromorphic devices based on their mixed transport characteristics.^[^ ^35^, ^36^, ^37^, ^38^, ^39^, ^40^, ^41^ ^]^ White et al. reported the first organic electrochemical transistor (OECT) using OMIEC polypyrrole (PPy) as the channel material in 1984,^[^ ^42^ ^]^ since when versatile research focusing on OECTs’ materials, structures, and applications has been conducted for decades.^[^ ^43^, ^44^, ^45^, ^46^, ^47^, ^48^, ^49^, ^50^, ^51^, ^52^ ^]^ OECTs operate in aqueous environments through the bulk doping of OMIECs and exhibit several key advantages, including high transconductance, excellent biocompatibility, and low operating voltage. These features make OECTs ideal platforms for biochemical and electrophysiological sensing interfaces with biological systems, and are particularly beneficial in biochemical sensors,^[^ ^53^, ^54^, ^55^, ^56^ ^]^ real‐time diagnostics,^[^ ^57^, ^58^, ^59^ ^]^ and implantable and wearable technologies.^[^ ^60^, ^61^, ^62^ ^]^ Furthermore, OECTs can replicate the process of ion‐driven regulation in biological systems thanks to the inherent ionic–electronic coupling of OMIECs. They are particularly well‐suited for developing devices that emulate neuronal and synaptic activities and integrate with biological systems since they offer compatibility with flexible and stretchable substrates, exhibit tunable ionic and electronic dynamics, support multimodal sensing, and operate at significantly low voltages (**Figure** 1).^[^ ^63^, ^64^, ^65^, ^66^, ^67^, ^68^, ^69^, ^70^, ^71^ ^]^ In neuron‐inspired designs, OECTs exploit dynamic conductance and capacitive effects to generate and encode spiking signals.^[^ ^72^, ^73^ ^]^ In artificial synapses, the OECT channel forms the synaptic cleft of an artificial chemical synapse; the gate electrode corresponds to the presynaptic membrane that receives stimulation; and the drain electrode simulates the postsynaptic receptor that collects postsynaptic current, providing a compelling analogy to neurotransmitter‐mediated synaptic transmission in biological systems.^[^ ^74^, ^75^ ^]^ Moreover, OMIECs demonstrate additional properties such as self‐healing^[^ ^76^, ^77^ ^]^ and physical evolvability,^[^ ^78^, ^79^ ^]^ further enhancing their suitability for adaptive and intelligent neuromorphic systems.

This review offers a comprehensive and state‐of‐the‐art vista of the rapidly advancing field of OECT‐based neuromorphic electronic devices and their applications. Initially, the fundamental operating mechanisms and electrical responses (both steady‐state and transient) of OECTs are elaborated. Then, recent advancements in organic electrochemical neurons (OECNs), organic electrochemical synapses (OECSs), and their integrated devices are discussed, with an emphasis on how they perform neuromorphic functionalities. Subsequently, various application scenarios are highlighted, including Boolean logic operations, reservoir computing (RC), Pavlovian conditioning, pattern recognition, biointerfaces, and flexible electronics. Finally, conclusions, remaining challenges, and future prospects for the development of OECT‐based neuromorphic devices are outlined. As a matter of fact, several previous reviews have discussed OMIECs for neuromorphic engineering, however, they generally included either OECNs^[^ ^63^, ^73^ ^]^ or OECSs,^[^ ^51^, ^52^, ^74^ ^]^ which ignored the integrated devices and their prevailing applications, since the first OECN was proposed only 3 years ago (2022)^[^ ^80^ ^]^ and the the first OECS was proposed 10 years ago (2015).^[^ ^81^ ^]^ Considering that both OECNs and OECSs have experienced tremendous advancements in the past 3 years, herein, we draw a thorough summary of up‐to‐date OECNs, OECSs, and their integrated applications, with a novel classification of neuromorphic computing (Boolean logic operations, RC, Pavlovian conditioning, and pattern recognition) and flexible biointerfaces (biointerfaces and flexible electronics).

## 2 Organic Electrochemical Transistors

### 2.1 Device Structure and Mechanism of OECTs

**Figure 2:**
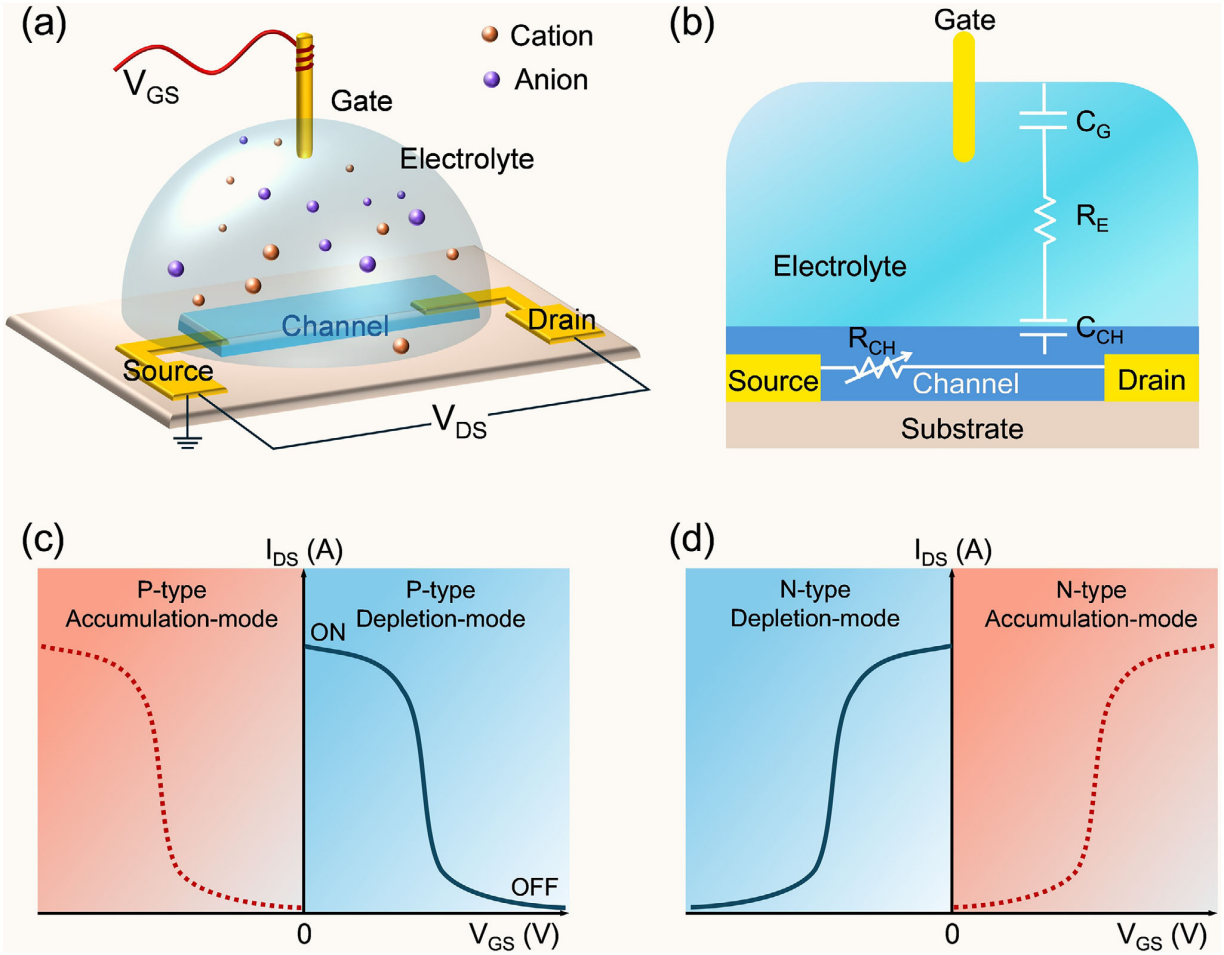
a) Schematic illustration of a typical OECT structure. b) Equivalent circuit of an OECT consisting of an ionic circuit (vertical) and an electronic circuit (lateral). c) Typical transfer curves of p‐type OECTs operating in depletion mode and accumulation mode. d) Typical transfer curves of n‐type OECTs operating in depletion mode and accumulation mode.

A typical OECT is in the form of a three‐terminal active device structure (**Figure** **2**a), primarily consisting of gate, source, and drain electrodes; an OMIEC channel deposited between the source and drain electrodes; an electrolyte connecting the channel and gate; and an encapsulation layer that reduces leakage current directly from the electrolyte to the source/drain electrodes, though this layer is not depicted in the schematic. Different from the channel materials in organic field‐effect transistors (OFETs) and electrolyte‐gated field‐effect transistors (EGFETs), OECT channels are generally made of OMIECs. Owing to the intrinsic capability for concurrent ionic and electronic transport, OMIEC‐based channels allow ions from the electrolyte to penetrate into the entire OMIEC layer, modulating the doping state (i.e., carrier concentration) and, consequently, the conductivity throughout the bulk channel rather than merely at the interfacial region under an applied gate voltage (*V_GS_*), which indicates tremendous amplification and high sensitivity to an input signal. In addition, the high volumetric capacitance of OMIEC channels in OECTs endows them with effective gate regulation and significantly lower operating voltages (less than 1 V) compared to those of their counterparts, such as conventional OFETs based on interfacial doping.

The fundamental electrical model of a typical OECT is divided into equivalent ionic (vertical) and electronic (lateral) circuits (Figure 2b).^[^ ^36^, ^83^, ^84^ ^]^ The electrochemical doping mechanism includes the mobile ion injection from the electrolyte into the entire OMIEC channel and subsequent diffusion, where the resistance of ion transport in the electrolyte is represented as the resistor *R_E_*, and the gate capacitance and channel capacitance are represented as the tandem capacitances *C_G_* and *C_CH_* in the ionic circuit, respectively.^[^ ^36^ ^]^ The channel capacitance comprises a parallel combination of multiple simplified plate capacitances, which are electric double layers (EDLs) formed by the injected ions and resident ions in the OMIEC channel.^[^ ^85^ ^]^ Strictly, an EDL is modelled by the Gouy–Chapman–Stern framework, encompassing a compact Helmholtz layer and a diffusing outer layer. Considering that gate voltage chiefly falls onto the relatively small capacitance, for efficient gating, the channel capacitance dominates the gate voltage drop, the gate capacitance should be significantly larger than the channel capacitance. This can be accomplished by substituting a nonpolarizable gate electrode (e.g., Ag/AgCl) that is ideally regarded as possessing the vanishing capacitance effect (i.e., infinite capacitance of EDL) with electrolyte owing to the presence of faradic current, in contrast to the polarizable gate electrode (e.g., Pt or Au). Leveraging the modulation of potential drop across either the gate capacitance or channel capacitance, various sensors for chemical and biological species have been extensively developed by either functionalizing the gate,^[^ ^53^, ^54^, ^60^, ^82^, ^86^, ^87^, ^88^ ^]^ channel,^[^ ^89^, ^90^, ^91^, ^92^, ^93^ ^]^ or electrolyte.^[^ ^94^, ^95^, ^96^ ^]^ Additionally, the variable resistor *R_CH_* in the electronic circuit, which is modulated by the ion injection under a gate voltage, clarifies the flowing resistance of electronic carriers in the channel under a voltage bias between drain and source (*V_DS_*).

Generally, based on the polarity of transport carriers in channel materials, OECTs can be mainly categorized into two kinds: p‐type OECTs, which support hole transport (Figure 2c), and n‐type OECTs, which support electron transport (Figure 2d). Additionally, depending on the intrinsic doping characteristics of channel materials, OECTs operate in two modes: depletion mode and accumulation mode. Figure 2c illustrates the transfer curves of p‐type OECTs operating in depletion mode (blue solid line) and accumulation mode (red dashed line), respectively. Taking the p‐type depletion‐mode OECT based on poly(3,4‐ethylenedioxythiophene):poly(styrene sulfonate) (PEDOT:PSS) as an example, in the absence of the gate voltage (*V_GS_* = 0 V), a high density of mobile holes is available for conducting current through the channel (*I_DS_*) under a drain voltage. When applying a positive gate voltage (*V_GS_* >0 V), cations in the electrolyte are repelled by the gate and then migrate into the OMIEC channel. This ionic motion compensates the anions in the PEDOT:PSS channel (PSS^−^), thereby reducing the density of mobile carriers responsible for conduction to maintain charge neutrality. On the contrary, a p‐type OECT operating in accumulation mode remains in an off‐state at zero gate bias due to a lack of sufficient intrinsic mobile carriers. Upon application of a negative gate voltage, anions from the electrolyte are injected into the channel, increasing the hole concentration and switching the device to the conductive on‐state. n‐type OECTs working in depletion mode and accumulation mode exhibit analogous operating mechanisms to their p‐type counterparts yet respond oppositely to gate bias, as shown by the blue solid and red dashed lines in Figure 2d, respectively.

### 2.2 Steady‐State and Transient Behaviors of OECTs

Bernards and Malliaras initially developed a systematic and comprehensive OECT model based on coupling the electronic charge transport mechanisms described by semiconductor physics with ionic transport dynamics described by electrochemistry stepwise in 2007, to quantitatively analyse the steady‐state and transient behaviors of OECTs.^[^ ^83^ ^]^ A p‐type depletion‐mode OECT was used as the representative example. According to the Bernards and Malliaras model, the expressions for drain current in the linear and saturation regimes are given by:^[^ ^83^ ^]^

(1) $$\begin{array}{c} \;I_{DS}=\mu C^{*}\frac{WT}{L}\left(V_{TH}-V_{GS}+\frac{V_{DS}}{2}\right)V_{DS},\;\left|V_{DS}\right|<\left|V_{GS}-V_{TH}\right|\\ \;I_{DS}=\mu C^{*}\frac{WT}{2L}{\left(V_{TH}-V_{GS}\right)}^{2},\;\left|V_{DS}\right|>\left|V_{GS}-V_{TH}\right|\\ \;V_{TH}=\frac{qp_{0}}{C^{*}}\; \end{array}$$

where the intrinsic properties of the channel material are determined by mobility (µ), volumetric capacitance (C*), and intrinsic carrier density (*p*_0_); *W*, *T*, and *L* are the geometric dimensions of the channel layer (i.e., width, thickness, and length, respectively); *V_TH_* is the threshold voltage, and *q* is the elementary charge. The electrical performance of OECTs is primarily determined by the characteristics of channel materials, the geometric dimensions of the channel, and the types and concentrations of electrolyte ions. To evaluate the performance of OECT devices, a key figure of merit is transconductance (*g_m_*), defined as the first derivative of the transfer curve, quantitatively representing the amplifying ability from input gate voltage to output drain current. Derived from the current expressions above, the expressions for transconductance in both the linear region and saturation regimes are given by:

(2) $$\begin{array}{c} \;g_{m}=\frac{\partial I_{DS}}{\partial V_{GS}}\;\\ \;g_{m}=-\mu C^{*}\frac{WT}{L}V_{DS},\;\left|V_{DS}\right|<\left|V_{GS}-V_{TH}\right|\\ \;g_{m}=\mu C^{*}\frac{WT}{L}\left(V_{TH}-V_{GS}\right),\;\left|V_{DS}\right|>\left|V_{GS}-V_{TH}\right| \end{array}$$

Although the expression shares a formal resemblance with that of traditional field‐effect transistors (FETs), a pivotal distinction lies in the replacement of the interfacial capacitance of the gate dielectric in FETs with the product of the volumetric capacitance and channel thickness in OECTs. The explicit presence of the channel thickness introduces an additional degree of freedom in device design, allowing for the realization of high transconductance values and effective modulation from gate voltage to drain current, which are the prevailing characteristics of OECTs compared to FETs.^[^ ^97^ ^]^

In addition to the steady‐state performance, the transient response of OECTs to dynamic input stimuli, such as changes in voltage or chemical species concentration, plays a critical role in understanding their operational dynamics, especially in applications where rapid response speed is essentially demanded. The Bernards and Malliaras model also offers an insightful view into the transient characteristics of OECTs, highlighting the potential effect of the channel transport time of the electronic circuit and the charging/discharging time of the ionic RC circuit, where the quantitative prediction of the transient drain current is given by:^[^ ^83^ ^]^

(3) $$\begin{array}{c} I_{DS}\;\left(t,\;V_{GS}\right)=I_{ss}\;\left(V_{GS}\right)+\Delta I_{ss}\left.\left(1-f\frac{\tau_{e}}{\tau_{i}}\right.\right)exp\left(-\frac{t}{\tau_{i}}\right)\\ \;\tau_{e}=\frac{L^{2}}{\mu V_{DS}}\;\\ \tau_{i}\;∼\;\frac{l}{\sqrt{C}} \end{array}$$

where *I_ss_* (*V_g_*) is the drain current when reaching steady state at an applied gate voltage and Δ *I_ss_* = *I_ss_* ( *V_g_* =  0) − *I_ss_* (*V_g_*); *f* is a proportionality constant describing the spatial non‐uniformity in the de‐doping process of the channel; τ_*e*_ represents the electronic transport time across the whole channel; τ_*i*_ represents the ionic transport time in the electrolyte path; *l* is the distance between the gate electrode and channel film; and *C* is the ionic concentration of the electrolyte. From the equivalent circuit model, the ionic transport time is generally dominated by the RC time constant of the electrolyte resistance and the ionic capacitances (i.e., gate capacitance and channel capacitance). The electrolyte resistance depends on a variety of factors, including the state, concentration, composition, ionic size, etc., of electrolyte,^[^ ^36^, ^98^ ^]^ while the ionic capacitance is relevant to the material characteristics and geometric dimensions of the gate and channel. For the sake of accelerating the ionic transport time, internal ion‐gated configurations offer a straightforward yet effective means by reducing the distance between the gate electrode and channel film.^[^ ^99^, ^100^ ^]^ With regard to the electronic transport time, it can be fruitfully improved by reducing the channel length and enhancing the carrier mobility in the OMIEC channel. Noticeably, when conventional planar OECTs reach the resolution limitation of standard fabrication techniques, vertical OECTs provide an alternative and effective path for further reducing the channel length and hence show benefits in obtaining high transconductance and outstanding response speed.^[^ ^100^, ^101^, ^102^ ^]^ The overall transient response time of an OECT device is determined by the combined effect of ionic and electronic dynamics.^[^ ^48^, ^82^, ^103^ ^]^ Focusing on the current expression, considering the relationship between τ_*e*_ and τ_*i*_, the transient response of drain current can be a monotonic decay (τ_*i*_ > *f*τ_*e*_) or a spike‐and‐recovery (τ_*i*_ < *f*τ_*e*_). The drain current monotonically relaxes from its starting to its final state in the first case, which happens when electronic transport exceeds ionic charging. The drain current exhibits a sudden spike before exponentially settling to its final value in the second case, which appears when ionic charging exceeds electronic transport. Several studies have validated these phenomena, highlighting the dynamic transient response of OECTs.^[^ ^97^, ^104^, ^105^, ^106^ ^]^ The latter spike‐shaped response is reminiscent of the sensing, encoding, processing, and transmission of electrical signals, serving as the basis for OECT‐based hardware neuromorphic devices.

### 2.3 Organic Mixed Ionic–Electronic Conductors for OECTs

**Figure 3:**
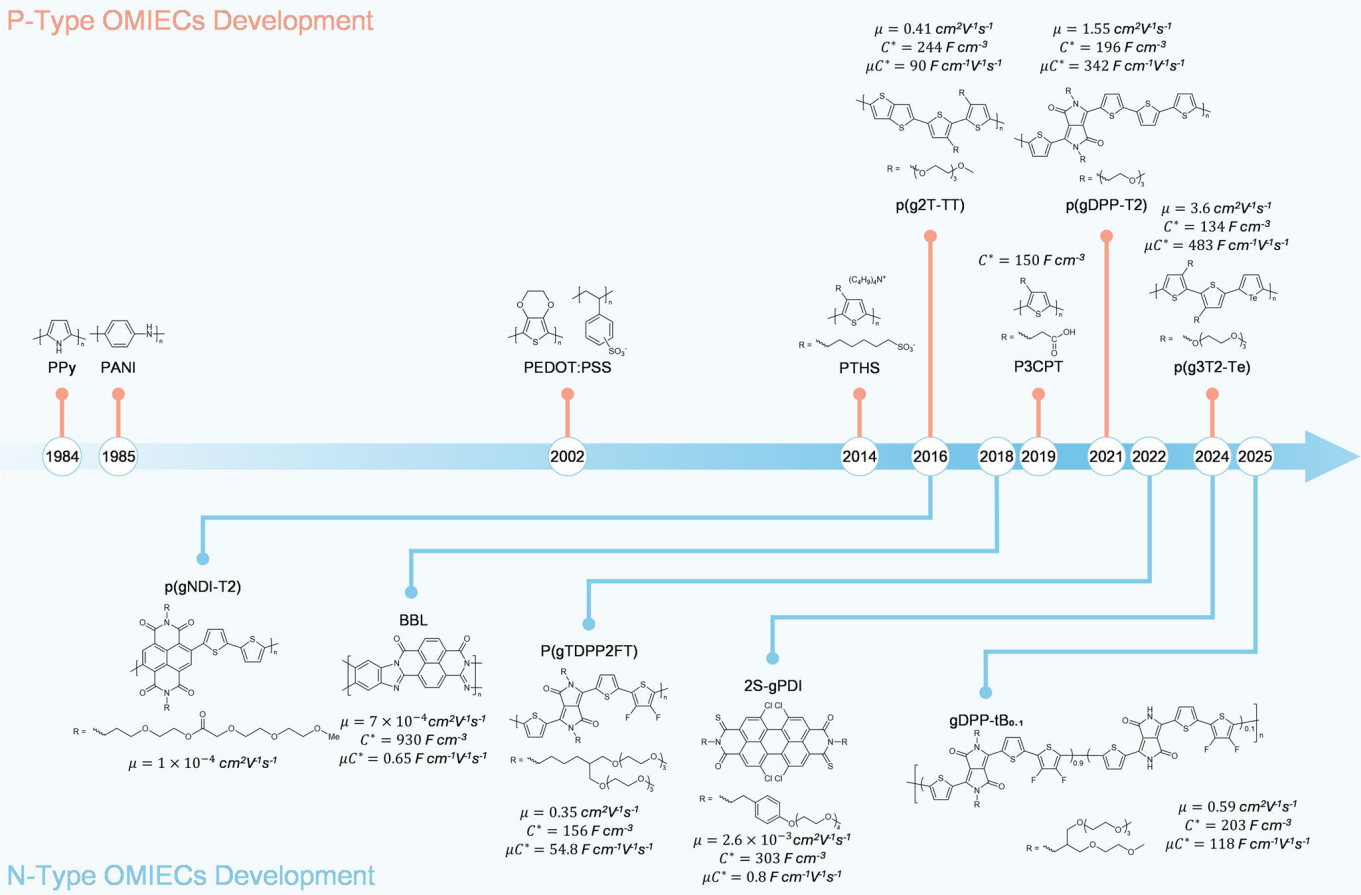
The material development of p‐ and n‐type OMIECs.

Considering that the overall performance of neuromorphic devices is dramatically determined by the channels, the material engineering of OMIECs exhibits innovative significance. OMIECs refers to a category of organic materials exhibiting both electronic (hole/electron) and ionic conduction capabilities, and the product of mobility and volumetric capacitance (µC*) represents the intrinsic performance of OMIECs that has been utilized as a benchmark since 2017.^[^ ^107^ ^]^ They present outstanding advantages, including tailorability, biocompatibility, and flexibility. As mentioned before, according to the polarity of transport carriers, OMIECs can be divided into p‐type ones (hole) and n‐type ones (electron). Their development begins with the early explorations of PPy^[^ ^42^ ^]^ and polyaniline (PANI),^[^ ^108^ ^]^ however, these materials suffer from low mobility and poor stability that inhibit their further progress. Later, PEDOT:PSS emerged in 2002 and remains one of the mainstream p‐type materials so far, benefiting from its high performance and commercial availability, which offers a template for subsequent material designs. OMIEC material designs mainly incorporate side chain engineering and backbone engineering.^[^ ^109^ ^]^ Although there have been versatile novel OMIECs via material engineering to date (**Figure** **3**), most of their compatible applications in neuromorphic devices are still under exploration.

#### 2.3.1 p‐Type Organic Mixed Ionic–Electronic Conductors

PEDOT:PSS represents a successful yet special p‐type OMIEC, which is stable in aqueous dispersion and formed by the Coulombic bond of electronic conductive PEDOT and ionic conductive PSS. The incorporation of PSS in the aqueous environment helps to resolve the insolubility problem of pristine PEDOT. Practically, PEDOT:PSS exhibits outstanding performance in both steady and transient states, in which its µC* reaches over 400 F cm^−1^V^−1^s^−1^ with treatment^[^ ^110^, ^111^ ^]^ and the response speed of its device reaches 20 µs.^[^ ^112^ ^]^ Based on these merits, PEDOT:PSS and its derivatives appeared as the most prevailing p‐type OMIEC for OECSs in the last 10 years. Nevertheless, it faces several limitations, such as depletion‐mode operation that causes excess power consumption and unavailability in complementary inverters, and PSS that induces restricted C* and corrosion risks. Therefore, abundant efforts have been devoted to expanding the OMIEC library to develop high‐performance OECTs and further relevant neuromorphic devices in the past several years.

Different from traditional organic electronic semiconductors, OMIECs universally possess hydrophilic side chains, facilitating bulk doping through the vacancy because ion injection and transport rely on the aqueous environment; however, excess swelling of OMIEC films might significantly hinder the transport of electronic carriers. Therefore, side chain engineering should balance the ion behaviours and electronic conductivity. To prevent the film from dissolving or delaminating in the aqueous environment, hydrophobic side chains^[^ ^113^, ^114^ ^]^ and crosslinkers^[^ ^115^, ^116^, ^117^ ^]^ might be considered along with hydrophilic side chains. The first attempt of side chain engineering dates back to 2014, when Inal et al. incorporated hydrophilic sulfonate groups into the conjugated polythiophene backbone (poly(6‐(thiophene‐3‐yl)hexane‐1‐sulfonate) tetrabutylammonium (PTHS) to facilitate bulk injection of ions into the OMIEC, drawing inspiration from the sulfonate groups of PEDOT:PSS.^[^ ^115^ ^]^ Later in 2016, Giovannitti et al. proposed a high‐performance p‐type OMIEC (poly(2‐(3,3′‐bis(2‐(2(2‐methoxyethoxy)ethoxy)ethoxy)‐[2,2′‐bithiophen]‐5‐yl) thieno [3,2‐b] thiophene)) p(g2T‐TT) and initiated the design route of glycolated side chains based on their hydrogen bonds with water molecules to replace previous alkyl and ionic chains.^[^ ^118^ ^]^ The glycolated side chains of p(g2T‐TT) significantly enhance the ion penetration with a high C*of 240 F cm^−^^3^ and acceptable swelling, which maintains the π–π stacking structure of the backbone, ensures high electronic conductivity, and removes the demand for crosslinkers and additives. Additionally, the coupling between glycolated side chains and water molecules helps to improve its response speed when operating, in which the OECT based on p(g2T‐TT) achieves a fast response speed of 67 µs.^[^ ^119^ ^]^ Since then, this route has become dominant, and there appears to be extensive research focusing on the length,^[^ ^120^ ^]^ structure,^[^ ^121^ ^]^ and distribution^[^ ^122^ ^]^ of the glycolated side chain. More recently, other hydrophilic groups, such as carboxyl and hydroxyl groups have been incorporated into side chains, in which poly [3‐(4‐carboxypropyl)‐thiophene] (P3CPT),^[^ ^123^ ^]^ poly[3‐(6‐hydroxy)hexylthiophene] (P3HHT),^[^ ^124^ ^]^ and poly(3‐{[2‐(2‐methoxyethoxy)ethoxy]methyl}thiophene‐2,5‐diyl) (P3MEEMT)^[^ ^125^ ^]^ represent the typical kinds. These materials exhibit reduced swelling and enhanced stability due to the moderate polarities of side chain groups, which showcase benefits for biological applications.

Backbone engineering chiefly contributes to polymer morphologies, π–π interactions, and energy levels to optimize electronic mobility and transport in OMIECs. Fixing backbone morphologies to promote coplanarity is generally achieved through intramolecular noncovalent interactions. For instance, the increased sulfur atoms on the backbone form O–S interactions with the oxygen atoms on the side chains of pgBTTT, which significantly facilitate rigidity of the backbone and enhance π–π interactions due to the reduced spacing with a high µC* of 563 F cm^−1^V^−1^s^−1^.^[^ ^126^ ^]^ Another means for improving π–π interactions refers to constructing a donor–acceptor (D–A) alternating arrangement as the backbone. Strong donors such as bithiophene (TT),^[^ ^126^ ^]^ benzo[1,2‐b:4,5‐b′] dithiophene (BDT),^[^ ^127^ ^]^ and 3,3′‐methoxybithiophene (MeOT2)^[^ ^128^, ^129^ ^]^ guarantee high mobilities on account of their wide conjugated ranges and strong delocalization, while weak acceptors such as diketopyrrolopyrrole (DPP)^[^ ^102^, ^130^, ^131^ ^]^ introduce locally polar sites to enhance ionic conductivity, concurrently improving the performance of µ and C*, in which p(gDPP‐T2) (342 F cm^−1^V^−1^s^−1^)^[^ ^132^ ^]^ and p(bgDPP‐MeOT2) (196 F cm^−1^V^−1^s^−1^)^[^ ^129^ ^]^ exhibit outstanding performance in µC*. Furthermore, heteroatom substitution on the backbone serves as an effective strategy for modulating both the intra‐ and intermolecular structures of polymeric mixed conductors. It plays a decisive role in controlling the dimensionality and orientation of polymer crystallites, thereby enabling precise tuning of mixed conduction properties. Among the oligoethylene glycol‐functionalized bithiophene chalcogenophene polymers, p(g3T2‐Te) demonstrated exceptional performance, achieving a µC* up to 483 F cm^−1^V^−1^s^−1^.^[^ ^133^, ^134^ ^]^

#### 2.3.2 n‐Type Organic Mixed Ionic–Electronic Conductors

In previous decades of development, n‐type OMIECs suffered from poor performance induced by the energy band misalignment, charge instability in the aqueous environment, and adverse effects between side chains and the backbone. Nevertheless, extensive research has been dedicated to developing high‐performance n‐type OMIECs since they are necessary in complementary inverters that construct the building blocks of integrated circuits. The first breakthrough dates back to 2016, when Giovannitti et al. reported poly(naphthalene‐1,4,5,8‐tetracarboxylic diimide‐co‐2,2'‐bithiophene) with ethylene glycol side chains (p(gNDI‐T2)), signifying the first n‐type OECT operating in water stably. The side chain engineering and backbone engineering of n‐type OMIECs are in a similar fashion to those of their p‐type counterparts.

Side chain engineering of n‐type OMIECs begins with p(gNDI‐T2). Promoting ion penetration, reducing excess swelling, and inhibiting adverse effects on the backbone illustrate the three pivotal principles when designing side chains. Appending glycolated side chains indeed promotes ion penetration; however, the oxygen atoms on side chains induce instability of the backbone and excess swelling. To solve these issues, Ohayon et al.^[^ ^135^ ^]^ and Maria et al.^[^ ^136^ ^]^ proposed an effective solution via inserting alkyl groups between the NDI backbone and glycolated side chains, which act as hydrophobic spacers to separate and decouple the NDI backbone and side chains. In a similar manner, Giovannitti et al. discovered the increased performance when they mixed the glycolated and alkyl groups as side chains in different proportions.^[^ ^137^ ^]^

Current backbone engineering of n‐type OMIECs primarily centers on the optimization of donor–acceptor (D–A) type polymers. The acceptor–acceptor (A–A) backbone, built from alternating electron‐deficient units, benefits from a fused structure that enhances stability and lowers the lowest unoccupied molecular orbital (LUMO) energy level. However, A–A polymers generally exhibit poor processability and weak n‐type OECT characteristics. Typical examples include PgNaN (µC* = 0.662 F cm^−1^V^−1^s^−1^)^[^ ^138^ ^]^ and p(g_7_NC_10_N) (µC* = 1.83 F cm^−1^V^−1^s^−1^).^[^ ^139^ ^]^ Conventional OMIECs based on D–A backbones are predominantly p‐type because donors behave more powerfully than acceptors. Incorporating halogen atoms with strong electronegativity (e.g., fluorine atom, F) or electrondrawing groups (e.g., cyanogroup, ─CN) into the backbone helps to transform the strong donor to a weak donor, which shifts LUMO downward and achieves n‐type doping. Li et al. reported a representative D–A n‐type OMIEC P(gTDPP2FT) with high performance by combining thiophene‐flanked diketopyrrolopyrrole (TDPP) and TT as the backbone, and incorporating two fluorine atoms on the thiophene with a notably high µC* of 42.2 F cm^−1^V^−1^s^−1^, comparable to its p‐type counterpart P(gTDPPT) with a µC* of 45.9 F cm^−1^V^−1^s^−1^.^[^ ^140^ ^]^ Improved cases come to gDPP‐tB_0_._1_^[^ ^141^ ^]^ and Pg_5_Tz‐5‐DPP^[^ ^142^ ^]^ with remarkable µC* values of 118 and 92.5 F cm^−1^V^−1^s^−1^, respectively. Feng et al. reported introducing cyanogroups into the thiophene–vinyl–thiophene donor to construct f‐BTI2g‐TVTCN and obtained a high µC* of 41.3 F cm^−1^V^−1^s^−1^.^[^ ^143^ ^]^ Lately, Ding et al. changed the backbone to link cyanogroups and synthesized S‐DTFMCN and B‐DTFMCN with moderate µC* values of 12.25 and 12.74 F cm^−1^V^−1^s^−1^, respectively.^[^ ^144^ ^]^ D–A n‐type backbone engineering has also witnessed significant improvements in 2DPP‐OD‐TEG^[^ ^145^ ^]^ and p(C2F‐V),^[^ ^146^ ^]^ etc. In addition, other design routes, such as hydrogen bond construction in the backbone^[^ ^147^ ^]^ and small molecule engineering^[^ ^148^, ^149^, ^150^, ^151^, ^152^ ^]^ also present effective performance improvements.

It is worth noting that ladder conjugated polymers composed of uninterrupted rings have also been developed and implemented in n‐type OECTs. Impressively, poly(benzimidazobenzophenanthroline) (BBL) represents a special n‐type OMIEC without hydrophilic side chains, which was initially reported by Sun et al. in 2018.^[^ ^153^ ^]^ The rigid and ladder backbone endows BBL with powerful π–π interactions and further efficient electronic transport, while the self‐assembling process affords nanopores for ion penetration and transport, removing the demand for side chains.^[^ ^154^ ^]^ In 2022, Wu et al. reported a high µC* of 25.9 F cm^−1^V^−1^s^−1^ of BBL_152_ and found that the increased molecular weight facilitates performance improvement, explained by the enhanced π–π interactions, optimized crystallinity, and inhibited scattering.^[^ ^155^ ^]^ Apart from material designs to improve the performance of BBL, film morphology manipulation through coating and post‐treatment also occupies a quite important position since the microstructures of experimental films are far from ideal cases.^[^ ^154^ ^]^ Benefiting from its leading performance, distinguishing antiampolarity, and stability, BBL has become the most prevailing n‐type OMIEC for OECT‐based complementary inverters and neuromorphic devices.

### 2.4 Advantages of OECTs for Neuromorphic Devices

In the vanguard of neuromorphic engineering, an interdisciplinary field merging neuroscience, computer science, electronics, and materials science, investigates neural phenomena and leverages electronic technologies to emulate, modulate, and interface with biological systems. It aims to address the limitations of traditional computing architectures in handling complex, parallel, and energy‐intensive tasks by leveraging biological inspiration. OECTs, grounded in organic semiconductors and operating based on the interaction between electronic carriers and ions that endow them with several salient advantages, have emerged as a pivotal technology within this domain.

Biocompatibility represents one of the most significant benefits of OECTs in neuromorphic engineering. The ability of a device to coexist peacefully with biological systems without causing any detrimental impact is referred to as its biocompatibility, requiring operational principles and physicochemical characteristics that are congruent with those of living tissues. From a materials standpoint, the organic constituents of OECTs (e.g., OMIECs and ion gels) exhibit mechanical properties (e.g., softness, elasticity, and stretchability) that closely resemble those of biological tissues.^[^ ^39^, ^47^, ^48^, ^49^, ^50^ ^]^ The flexible structure of OECT might mitigate mechanical damage to biological tissues (e.g., brain tissues and nerve fibers) during implantation or connection, as well as the risk of inflammatory reactions or tissue fibrosis brought on by long‐term implantation, in contrast to the rigidity of conventional silicon‐based semiconductor devices. OECT‐based neuromorphic sensors, for example, may be designed as peripheral equipment or be implanted to fit around the brain or other organs, offering a less invasive and more natural approach of tracking and processing biological signals as biointerfaces.^[^ ^75^, ^79^, ^80^, ^112^, ^156^, ^157^, ^158^ ^]^ Moreover, the structure and ionic‐electronic coupling characteristics of OMIECs bear strong resemblance to the structure and operating process of biological systems, which rely on ion flux and chemical signaling. However, existing neuromorphic devices generally rely on electrical current and action potential communication, ignoring the intrinsic working mechanisms of the ion flow across neuron cell membranes and the neurotransmitter delivery within synapses.^[^ ^47^, ^49^ ^]^ Thus, bridging this gap has motivated the development of biochemically mediated neuromorphic devices that incorporate neurotransmitter dynamics and biorelevant ionic interactions, aiming to more faithfully replicate neuron‐to‐neuron communication and achieve biorealistic applications.^[^ ^74^, ^75^, ^159^, ^160^, ^161^, ^162^ ^]^ Benefiting from the biorealistic biochemical sensing, neuromorphic devices may achieve compact internal integration and multimodal perception.^[^ ^157^, ^163^ ^]^ Furthermore, thanks to the tunability of ionic–electronic coupling in OECTs through channel,^[^ ^164^ ^]^ electrolyte,^[^ ^165^ ^]^ and gate engineering,^[^ ^166^ ^]^ the conductance state can be permanently and steadily modulated into multiple values, providing the foundation for artificial synapses.

Another pivotal advantage of OECTs lies in their exceptional processability and mechanical flexibility. Unlike rigid silicon‐based electronics, which typically require complex fabrication process such as vacuum deposition and photolithographic etching, organic semiconductors used in OECTs can be readily deposited on a wide variety of substrates, including flexible plastics,^[^ ^167^, ^168^, ^169^ ^]^ textiles,^[^ ^170^, ^171^, ^172^ ^]^ fibers,^[^ ^173^, ^174^ ^]^ paper,^[^ ^175^, ^176^ ^]^ thin films,^[^ ^177^ ^]^ etc., through solution‐processing techniques such as spin‐coating, inkjet printing,^[^ ^45^, ^178^, ^179^, ^180^ ^]^ and screen printing,^[^ ^45^, ^80^, ^181^, ^182^, ^183^ ^]^ etc. These low‐cost and scalable manufacturing techniques facilitate the production of large‐area arrays and circuits of OECTs with high precision and reproducibility.^[^ ^184^ ^]^ From the perspective of channel materials, organic molecules in the OMIECs utilized in OECTs can bend and rotate in response to external mechanical deformation such as stretching and bending, endowing them with a high degree of flexibility.^[^ ^37^ ^]^ The employment of a liquid or solid electrolyte further enhances their flexibility, complementing the mechanical compliance of the polymer channels.^[^ ^185^, ^186^, ^187^, ^188^, ^189^, ^190^ ^]^ This high degree of flexibility is particularly crucial for applications involving conformal integration with curved or soft biological tissues or irregularly shaped mechanical systems.^[^ ^61^, ^177^, ^191^ ^]^

In general, OECTs are appealing to neuromorphic applications because of their inherently low operating voltages and reduced power consumption per switching event. In terms of the organic materials utilized in OECTs, thanks to their bulk doping characteristics, OMIECs exhibit high volumetric capacitance, thereby facilitating effective gating at low voltages. In other words, redox doping and de‐doping in polymeric materials is a connaturally low activation‐energy process, where the effortlessness of reversible ion exchange is supported by the space between polymer chains, or the free volume, allowing for switching with low applied voltages and energy consumption.^[^ ^64^ ^]^ This low‐voltage operation is crucial for preserving the structural integrity of biological and chemical analytes, particularly in biological integrated applications where high voltages might lead to membrane rupture or cell death during the interrogation of living cells and tissues.^[^ ^49^, ^50^, ^192^ ^]^ When it comes to the field of biointerface, the low working voltage not only ensures stable performance and significantly mitigates the potential risk of injury to the body of humans but also makes OECTs attractive to electronic devices with low power consumption and long‐term operation. In 2023, Huang et al. fabricated a complementary inverter composed of vertically structured p‐ and n‐type OECTs operating at only 0.7 V, with remarkable switching stability exceeding 50 000 cycles.^[^ ^102^ ^]^ The benefits of OECTs in terms of operating voltage and power consumption are much more noteworthy when considering energy‐efficient neuromorphic devices and integrated systems. In 2017, Burgt et al. reported a dual‐mode OECS operating under an ultralow switching voltage of 0.5 mV and energy consumption of 10 pJ per switching on a 1000 µm^2^ device, comparable to the energy consumption density in biological synapses.^[^ ^164^ ^]^

## 3 OECT‐Based Neuromorphic Devices

### 3.1 Organic Electrochemical Neurons

In coming to biological neuron behaviors, it uses sensory receptors that are sensitive to diverse external stimuli to convert sensory information into neural spikes. Upon receiving a sufficiently strong sensory stimulus, the ion channels in the cell membrane are activated to propagate the action potential. Benefiting from the tunable conductance of OMIEC channels in response to chemical or biological mediations, OECTs are capable of serving as artificial counterparts that respond to sensory information applied at the gate. With differing degrees of fidelity to the sophisticated encoding and processing of biological neurons, several hardware‐based spiking neuron models have been developed to elucidate spike generation mechanisms, including the leaky integrate‐and‐fire (LIF) model and the Hodgkin–Huxley (HH) model.^[^ ^193^ ^]^ All of these models, in spite of their structural differences, emulate the classical neuronal behavior wherein input currents are integrated until a threshold membrane potential is reached, triggering output spikes transmitted to subsequent neurons.^[^ ^73^ ^]^ Reported OECT‐based neuron implementations can be broadly classified into four categories based on their circuit models: LIF‐OECNs, organic electrochemical nonlinear device‐based OECNs (OEND‐OECNs), conductance‐based OECNs (C‐OECNs), and single‐transistor OECNs (1T‐OECNs), each possessing distinct advantages and mimicking various aspects of ion channel dynamics (**Table** **1**).

**Table 1:** Summary of OECNs and their applications.

Model	Channel material	Electrolyte	Operating frequency	Power consumption	Application	Refs.
LIF‐OECN (A‐H)	p‐type: P(g_4_2T‐T) n‐type: BBL	NaCl	46–274 mHz	15 µW (inverter)	Biointerface with venus flytraps	[80]
OEND‐OECN	T1: PEDOT:PSS T2: p(g2T‐TT)	NaCl	6–40 Hz	24 µW, 57 nJ per spike	Biointerface with Caco‐2 epithelial cells, sensing of Na+, K+, and dopamine	[156]
LIF‐OECN (A‐H)	p‐type: PBBT‐Me n‐type: BBL	NaCl	1–2 Hz	10 µW (inverter)	None	[195]
C‐OECN	BBL	NaCl	5–100 Hz	60 µW, 175 nJ per spike	Biointerface with rat vagus nerves, sensing of Na+, acetylcholine, dopamine, and glutamine	[198]
C‐OECN	BBL/PEDOT bilayer	PBS	0–20 Hz	NA	Retina‐inspired processing system of sensing light intensity, Boolean logic operations	[199]
LIF‐OECN (A‐H)	Inverter: p(C4‐T2‐C0‐EG) (ambipolar) Resetting Transistor: P‐3O	PBS	0.09–0.25 Hz	NA	Retina‐inspired processing system of sensing light intensity, dopamine, and serotonin	[157]
LIF‐OECN (A‐H)	p‐type: gDPP‐g2T n‐type: Homo‐gDPPTz	PBS	0.13–147.1 Hz	15 nW (inverter)	Integration with pressure sensors and strain sensors to realize pattern recognitions	[131]
LIF‐OECN (A‐H)	p‐type: PTBT‐P n‐type: BBL/PEI (GIBS)	NaCl	22–50 Hz	NA	Classical conditioning of mouse leg muscles, multimodal sensing of light, temperature, and Ca^2^⁺	[196]
1T‐OECN	BBL	NaCl NH_4_Cl NH_4_Br	1.4–32 Hz	16.43 nW, 4.7 nJ per spike.	Biointerface with rat primary cortical neurons, integration with pressure sensors, Boolean logic operations	[72]

#### 3.1.1 Leaky Integrate‐and‐Fire Organic Electrochemical Neurons

**Figure 4:**
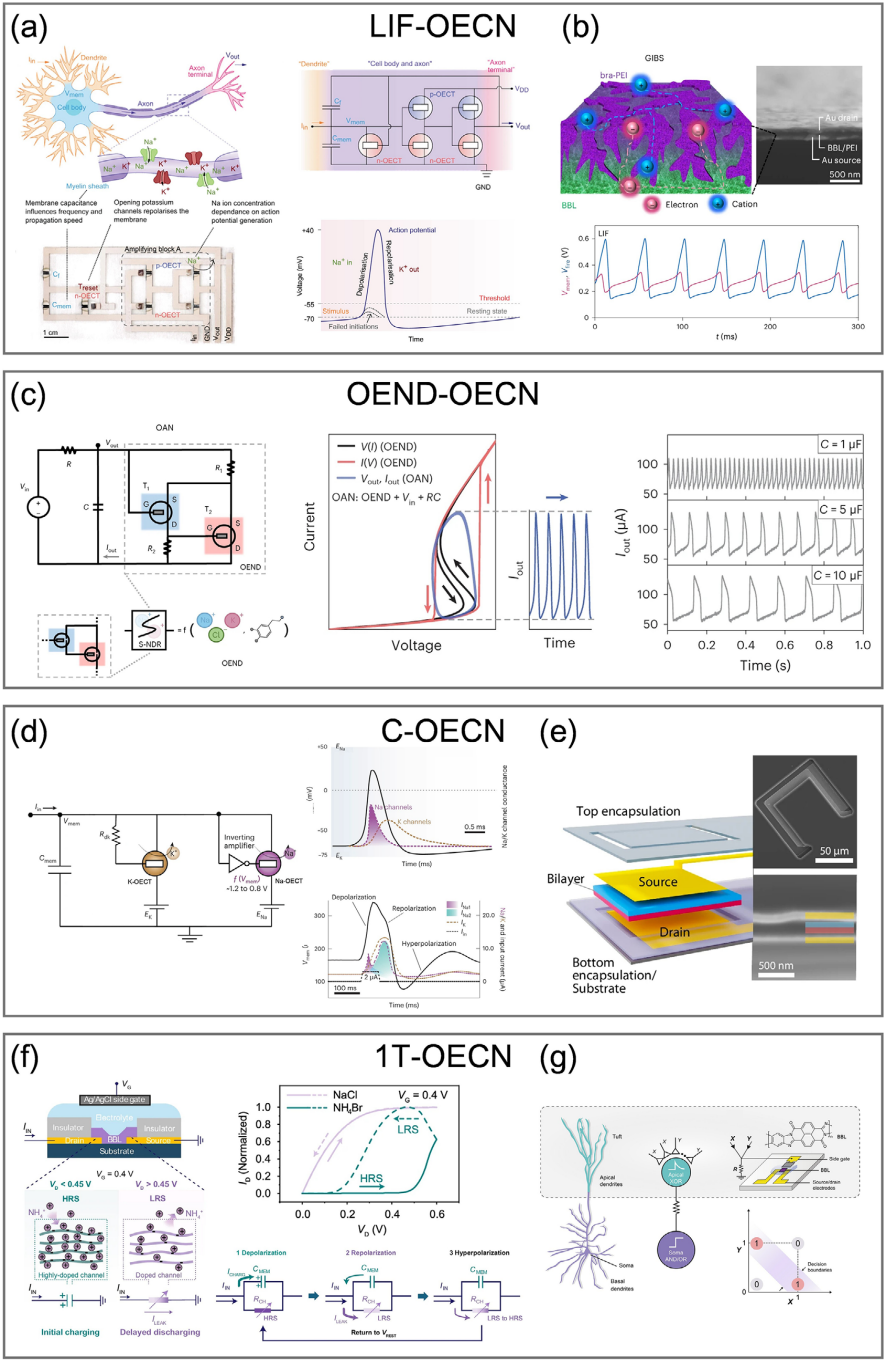
OECNs based on the four neuron models. a) The first experimental LIF‐OECN based on the A–H model. Reproduced with permission.^[^ ^80^ ^]^ Copyright 2022, Springer Nature. b) High‐performance LIF‐OECNs with a GIBS composed of BBL and PEI. Reproduced with permission.^[^ ^196^ ^]^ Copyright 2025, Springer Nature. c) OEND‐OECNs with S‐NDR characteristics consisting of two p‐type depletion‐mode and accumulation‐mode OECTs. Reproduced with permission.^[^ ^156^ ^]^ Copyright 2022, Springer Nature. d) C‐OECNs consisting of a K‐OECT and a Na‐OECT. Reproduced with permission.^[^ ^198^ ^]^ Copyright 2023, Springer Nature. e) C‐OECNs with a vertical bilayer structure composed of BBL and PEDOT:PSS. Reproduced with permission.^[^ ^199^ ^]^ Copyright 2024, Springer Nature. f) 1T‐OECNs based on antiambipolar BBL. Reproduced with permission.^[^ ^72^ ^]^ Copyright 2025, Springer Nature. g) Dendritic 1T‐OECNs. Reproduced with permission.^[^ ^203^ ^]^ Copyright 2025, American Association for the Advancement of Science.

The widely adopted LIF‐OECN features a straightforward operational mechanism that replicates the essential process of signal integration and threshold‐triggered activation found in biological neurons, while omitting more complex neural dynamics. In 2022, Harikesh et al. experimentally constructed a LIF‐OECN by selecting the Axon–Hillock (A–H) circuit for the first time, a compact yet effective model appropriate for event‐triggered perception and SNNs (**Figure** **4**a).^[^ ^80^ ^]^ The circuit consists of two capacitances: membrane capacitance *C_mem_* and feedback capacitance *C_f_* manipulating the process of charging and discharging, n‐type OECTs based on BBL, and p‐type OECTs based on glycolated polythiophene (P(g_4_2T−T)), in which the performance of complementary OECTs is balanced through careful adjustment of the channel layer thickness. In biological neurons, owing to the insulation and barrier characteristics of the lipid cell membranes, apart from the ion channels, the intracellular resting potential maintains an excess negative potential close to −75 mV, which is the potassium ion (K^+^) Nernst potential due to its dominant permeability at rest. Upon continuous stimulation, voltage‐gated sodium ion (Na⁺) channels open rapidly as the membrane depolarizes toward the action potential threshold, allowing an influx of Na⁺ ions in a positive feedback loop and resulting in the membrane potential close to the sodium Nernst potential (+55 mV). Repolarization subsequently restores the membrane to its resting state as sodium channels close and potassium channels open, causing an excess outflow of potassium ions. The action potential is transmitted when these events are repeated in the neighboring membrane areas. Analogously, in the LIF‐OECN, membrane capacitance integrates input current until it reaches the threshold voltage of the amplifying block, producing a spike at the output. Upon reaching the peak output, the n‐type resetting OECT initiates discharging and gradually returns the circuit to its resting state. The oscillation frequency is predominantly governed by the input current, the values of membrane capacitance and feedback capacitance, and the performance of both the n‐ and p‐type OECTs. The OECN circuit functions at a low operating voltage of 0.6 V with a maximum dynamic inverter power consumption of 15 µW, which is considerably lower than that of its OFET‐based counterpart (40 µW),^[^ ^194^ ^]^ with the amplifying module accounting for the majority of power consumption.

The operating range of OECNs is primarily limited by the dynamic performance of OECTs, given that the capacitance and input current in the A–H model can be flexibly modulated to increase the frequency of output spikes. Recently, another LIF‐OECN based on the A‐H model was proposed by exploiting vertical OECTs with poly(bis(2‐[2‐(2‐methoxyethoxy)ethoxy]ethyl)‐3,6‐di(thiazol‐2‐yl)pyrrolo[3,4‐c]pyrrole1,4(2H,5H)‐dione) (Homo‐gDPPTz) serving as the n‐type material and (gDPP‐g2T) serving as the p‐type material.^[^ ^131^ ^]^ Thanks to the fast response enabled by the vertical structure and the outstanding and well‐matched performance of the n‐ and p‐type complementary channels, the inverter response frequency can reach 500 Hz, and the resulting OECN operating frequency ranges from 0.13 to 147.1 Hz, perfectly encompassing the frequency range of neural activities and dramatically broadening the frequency range of A–H model‐based OECNs (several Hz).^[^ ^79^, ^80^, ^195^ ^]^

Historically, the performance of n‐type materials has always lagged behind that of p‐type counterparts, constraining the applications of organic inverters. Nonetheless, recent advances have led to significant improvements in n‐type material properties, with BBL playing an instrumental role in the development of neuromorphic devices. A recent study proposed a LIF‐OECN with a vertical gradient‐intermixed bicontinuous structure (GIBS) composed of BBL and polyethylenimine (PEI), formed via sequential deposition as the n‐type channel (Figure 4b).^[^ ^196^ ^]^ In GIBS, BBL serves as the electronic conductor, while PEI serves as the ionic conductor. The hydrophilic amino groups in PEI suppress electrochemical swelling and reduce ion diffusion distance, optimizing the response time to 27 µs, whose speed exceeds 100 times faster than that of conventional n‐type materials (e.g., p(gNDI‐gT2)^[^ ^197^ ^]^ and gNR^[^ ^150^ ^]^). Another material engineering route to performance improvement involves the synthesis of ladder polymers.^[^ ^195^ ^]^ These material engineering efforts significantly improve the performance and stability of the channel, enabling improved matching and enhanced operation of inverters and OECNs.

#### 3.1.2 Organic Electrochemical Nonlinear Device‐Based Organic Electrochemical Neurons

Unlike LIF‐OECNs, which require integration with peripheral sensory devices for input, OEND‐OECNs provide an alternative architecture that integrates sensing and signal encoding internally, resulting in a more compact and biorealistic platform. The OEND architecture, composed of two OECTs and two feedback resistances, exhibits S‐shaped negative differential resistance (S‐NDR) characteristics under current sweep conditions, providing the basis for the interaction with the capacitance and current spikes generation at the output. In 2022, Sarkar et al. employed PEDOT:PSS as the p‐type depletion‐mode channel material and p(g2T‐TT) as the p‐type accumulation‐mode channel material to construct the two OECTs in cascade to form the S‐NDR characteristics (Figure 4c).^[^ ^156^ ^]^ The frequency of the output spikes can be modulated by the circuit capacitance and the input voltage source. Regarding sensing mechanisms, the spiking frequency is highly sensitive to the ion concentration and biological molecules because these significantly affect the threshold voltages and response times of the two OECTs, altering the charging and discharging dynamics. This biochemically mediated mechanism endows OEND‐OECNs with biorealistic functions to vividly imitate versatile biological neuronal behaviors. However, the system operates at a relatively high constant voltage (1.75 V) and generates current spikes instead of voltage spikes, which hinder the integration with biological systems and other neuromorphic devices.

#### 3.1.3 Conductance‐Based Organic Electrochemical Neurons

To further emulate the functional mechanisms of biological potassium and sodium channels separately, as described by the HH model, a C‐OECN was proposed by Harikesh et al. in 2023 (Figure 4d).^[^ ^198^ ^]^ Despite its widespread applications in n‐type OECTs, BBL exhibits antiambipolar properties with an inverted V‐shaped transfer curve (Gaussian shape). By taking advantage of the stable and reversible antiambipolarity of BBL, the simulation of the activation and inactivation process of ion channels can be achieved. The immediate sodium channel response and delayed potassium channel response are accomplished by manipulating the thickness of active BBL layers, adjusting the threshold voltage of the K‐OECT, and incorporating an inverting amplifier in the Na‐OECT pathway. Since the threshold voltages of the two OECTs are highly sensitive to biochemicals, these in turn significantly modulate the frequency of output voltage spikes, highlighting the potential of such systems in biointerface applications. This model is capable of reproducing 15 out of 20 neuronal features, exhibits low energy consumption of 175 nJ per spike, and operates at a maximum frequency of 100 Hz, which aligns with the typical firing range of biological neurons. Subsequently, Laswick et al. proposed a material engineering approach by optimizing the channel into a vertical bilayer structure composed of BBL and PEDOT:PSS, exhibiting a broader frequency range compared to single BBL devices (Figure 4e).^[^ ^199^ ^]^ Nevertheless, the necessity of the inverting amplifier, along with increased circuit complexity and the demanding stability requirements within the operating range, hampers the applicability of the model in practical biorealistic implementations.

#### 3.1.4 Single‐Transistor Organic Electrochemical Neurons

More recently, a BBL‐based 1T‐OECN drawing inspiration from memristors was demonstrated by Ji et al (Figure 4f).^[^ ^72^ ^]^ To imitate the features of memristors, the device achieves hysteretic switching between the high resistance state (HRS) and the low resistance state (LRS) by leveraging ion‐tunable antiambipolar features and prominent asymmetric transient responses of doping (forward scanning) and de‐doping (backward scanning) processes, thereby resembling the delayed activation of potassium channels observed in biological neurons. Beyond its memristive characteristics, this design leverages the intrinsic capacitance of the channel to integrate the input current at the drain and generate oscillating output spikes. The spiking frequency is modulated by both the magnitude of the input current and the electrolyte properties (e.g., species and concentration). In contrast to existing LIF‐OECNs that typically operate by integrating the voltage at the gate, this design presents a brand‐new alternative path for generating spikes based on current integration at the drain side. To further imitate the delayed activation of potassium channels in biological neurons, ammonium bromide (NH_4_Br) electrolyte is selected instead of the conventionally used sodium chloride (NaCl) or potassium chloride (KCl) electrolyte because the hydrogen bond between the ammonium ion (NH_4_^+^) and BBL greatly extends the response time of the de‐doping process. This design significantly simplifies traditional artificial neuron models, reduces the footprint of OECNs with a compact size of 177 µm^2^ and an integration density of 62 500 neurons cm^−2^, exceeding human cortex neuron density, and restrains the energy consumption to 4.7 nJ per spike, demonstrating its potential for high‐density integration of neuromorphic systems with biological applications. In addition to the abovementioned transforming direct current signals into oscillating voltage signals and encoding current amplitudes into voltage frequencies operated on somas and axons, the dendrites in the neuron act as receiving signals from multiple inputs and conducting judgment, which generally corresponds to the multi‐gate structures of OECTs.^[^ ^200^, ^201^, ^202^ ^]^ Harikesh et al. proposed a dendritic OECN achieving the Gaussian nonlinear activation of dendritic calcium channels with the same BBL‐based 1T‐OECN (Figure 4g).^[^ ^203^ ^]^ Thanks to the antiambipolar characteristics of BBL, which indicate that only moderate gate voltages open the channel, while low and high gate voltages maintain the channel closed, the Gaussian nonlinear activation can be accomplished in the form of XOR operation. This demonstration not only removes the need for multilayer neural networks to achieve dendritic XOR operation with a single OECT but also significantly reduces the complexity and power consumption of neuromorphic circuits.

### 3.2 Organic Electrochemical Synapses

A synapse is a specialized structure that connects neurons or neurons with other cells to transmit information, which is an essential component for signal transmission and functional integration within the neural system. Synaptic plasticity, referring to the ability of synapses to vary their transmission efficiency or synaptic weight in response to the input activity, underlies pivotal neural processes such as learning and memory. Artificial synapses are devices and technologies constructed for emulating synapses and performing synaptic plasticity, including short‐term plasticity (STP, milliseconds to seconds)^[^ ^204^ ^]^ and long‐term plasticity (LTP, over hours)^[^ ^205^ ^]^ for the transmission, encoding, and filtering of neural signals.^[^ ^206^ ^]^ Specifically, for OECSs, STP is associated with the transient occupancy of ions in OMIEC channels, whereas LTP deals with prolonged ion retention. While the mechanisms of learning and memory are closely linked to LTP, STP supports various dynamic computational functions in the brain.^[^ ^64^, ^207^ ^]^ Artificial synapses primarily emulate chemical synapses, relying on the neurotransmitters to transfer information with high specificity and complexity, while biological synapses incorporate both chemical and electrical synapses.^[^ ^64^, ^65^, ^67^ ^]^ At present, extensive research has explored both theoretical models^[^ ^208^, ^209^ ^]^ and biomimetic implementations^[^ ^13^, ^78^, ^79^, ^210^ ^]^ that replicate synaptic behaviors based on STP and LTP (**Table** **2**).

**Table 2:** Summary of OECSs and their salient features.

Synaptic plasticity	Channel material	Electrolyte	Gate material	LTP retention time	LTP state	Operating volatge [V]	Energy consumption [pJ per spike]	Salient feature	Refs.
STP	PEDOT:PSS	KCl	Au	/	/	0.5	NA	First OECS	[81]
STP LTP	PEDOT:PTHF	KCl	Ag/AgCl	Several hours	NA	0.5	NA	First OECS with LTP	[211]
STP	PEDOT:PSS	NaCl	Ag/AgCl	/	/	0.3	NA	Global and synchronous gating with soft connections	[210]
STP LTP STDP	PEDOT:PSS/PEI	NaCl KCl Nafion	PEDOT:PSS	Over 25 h	500	1	10	Decoupling the writing and reading process	[164]
STP	PEDOT:PSS	PBS	Pt	NA	NA	0.5	2	Dynamic reconfiguration	[202]
STP LTP STDP	PETE‐S	NaCl	Ag/AgCl	Several months	NA	−0.5, −2	1	In situ evolution and transition	[78]
STP LTP	PEDOT:PSS	LiCl NaCl CH_3_COONa	Multilayer graphene	Several hours	100	1	NA	Gate engineering	[166]
STP LTP	PTIIG‐Np	[EMIM][TFSI]/ PS‐PMMA‐PS	Same with electrolyte	230.1 s	NA	−2.5	NA	Modulating plasticity by crystallinity	[220]
STP LTP	PDPP	[EMIM][TFSI]	Same with electrolyte	Over 10 min	NA	−0.4	0.1	First vertical OECS with high current density	[225]
STP LTP	PEDOT:PSS	Cell culture media	PEDOT:PSS	Over 24 h	NA	0.3	NA	Neurotransmitter‐mediated LTP	[75]
STP	PEDOT:PSS	[Li][TFSI]	Au	/	/	−0.5	NA	Inserting silver nanowires	[212]
STP LTP	P3CT	[EMIM][TFSI]	Same with electrolyte	Over 1000 s	5	−1.5	2	Supporting spatiotemporal iterative learning	[226]
STP	PEDOT:PF6	PBS	Au	/	/	1	NA	Channel electropolymerization	[227]
STP LTP	PEDOT:PSS/ PAAm	PBS	Ag/AgCl	NA	NA	0.7	0.113	Nanofibrous channel	[173]
STP	PEDOT:PSS	NaCl	PEDOT:PSS	/	/	0.5	2	Post‐fabrication control with dual gates	[214]
STP LTP STDP	PEDOT:Tos/ PTHF	NaCl	Ag/AgCl	Over 200 min	NA	0.6	NA	Ion trapping	[228]
STP LTP	PEDOT:PSS	NaCl	Au	NA	NA	7	NA	Inserting a Nafion membrane	[229]
STP	PEDOT:PSS	Tris and NaCl	PEDOT:PSS	/	/	0.3	NA	Inserting a supported lipid bilayer	[230]
STP LTP STDP	PETE‐PC	NaCl and ETE‐PC solution	Ag/AgCl	Over 1000 s	150	−0.6	NA	Channel electropolymerization	[80]
STP LTP	PEDOT:PSS	PSS:Na and EDOT	PEDOT:PSS	Over 2 months	NA	0.7	3000	Gate electropolymerization	[231]
STP LTP	PETE‐S	NaCl and ETE‐S	Ag/AgCl	Over 9.5 h	NA	0.5	NA	Channel electropolymerization	[79]
STP	PEDOT:PSS	[MTEOA][MeOSO_3_]	Au	/	/	−2	NA	Self‐healing electrolyte	[232]
STP LTP	P3HT:PCBM	LiTFSI LiPF_6_ TBACl	Au	Over 2 h	280	−0.6	NA	Photon modulation	[222]
STP LTP	PEDOT:PSS	Chemically sensitive ion gel	Au	Several hundred seconds	NA	1.3	NA	Chemical modulation	[224]
STP LTP STDP	PTBT‐p	[EMIM][TFSI]	Au	Over 10 000 s	1,024	1.5	0.3	Crystallinity modulation	[233]
STP LTP	PEDOT:PSS	PBS	Bi_2_S_3_ and hydrogel	Over 2000 s	NA	0	NA	Light modulation without gate voltage	[234]
STP LTP	P3HT/SEBS	[EMIM][TFSI]/ PVDF‐HFP	Au	NA	100	3	NA	Intrinsic stretchability	[235]
STP	PEDOT:PSS	NaCl	Ag ink	Over 10 s	NA	0.3	NA	Neurotransmitter‐mediated LTP	[159]
STP	DPPT‐TT	Chitosan	NA	/	/	3	NA	All‐solid‐state OECS	[236]
STP	PEDOT:PSS	Shewanella basal medium	PEDOT:PSS	/	/	0.5	NA	Extracellular electron transfer of a bacterium	[163]
STP LTP	P3HT:APT	[BMIM][TFSI]/ PVDF‐TrFE	Au	Over 3000 s	NA	−2	NA	Crystallinity modulation and ion trapping	[217]
STP LTP	p(gDPP‐V)	NaCl	Ag/AgCl	Over 40 s	NA	NA	NA	Ambipolar transition between STP and LTP	[130]
STP LTP	PEDOT:PSS	KCl and MOPS buffer solution	Ag/AgCl	10–500 s	NA	0.1	NA	Droplet bilayer strategy	[165]
STP LTP	gNR‐Pr	Na3Cit gel	Au	Over 800 s	256	1.8	0.047	Conformation and side‐chain engineering	[237]
STP LTP	P3HT	[BMIM][TFSI]	Au	Over 130 s	69	−4	NA	Inserting a MOF Layer (Ni‐HHTP)	[219]
STP LTP	p(g2T‐T)	Ion‐gel	Ag/AgCl	NA	NA	−0.5	NA	Photo‐cross‐linking strategy	[238]
STP	PEDOT:PSS	Ion‐gel	Ag/AgCl	/	/	0.7	NA	Integration with OECN	[131]
STP LTP	BBL/PEI	[EMIM][TFSI]/ PVDF‐HFP	Au	Over 1000 s	512	2	NA	Gradient‐intermixed bicontinuous structure	[196]
STP LTP	PEDOT:Tos/PTHF	NaCl	Ag/AgCl	NA	1000	0.6	NA	Dual‐mode memory	[239]
STP LTP	PEDOT:PSS	[EMIM][EtSO_4_]	Au	NA	100	3.5	NA	Film morphology modulation	[240]
STP LTP	P3gCPDT‐1gT2	Hydrogel	PEDOT:PSS	Over 300 s	512	−3	NA	All‐polymer configuration	[241]
STP LTP STDP	p(C2F‐z)	NaCl	Ag/AgCl	Over 340 s	512	0.4	NA	Light modulation in UV−VIS−NIR	[242]
STP LTP STDP	P3HT:Y6	[BMIM][TFSI]	Ag	Over 30 min	NA	−0.2	NA	Light modulation in UV−VIS−NIR	[243]

#### 3.2.1 Short‐Term Plasticity

**Figure 5:**
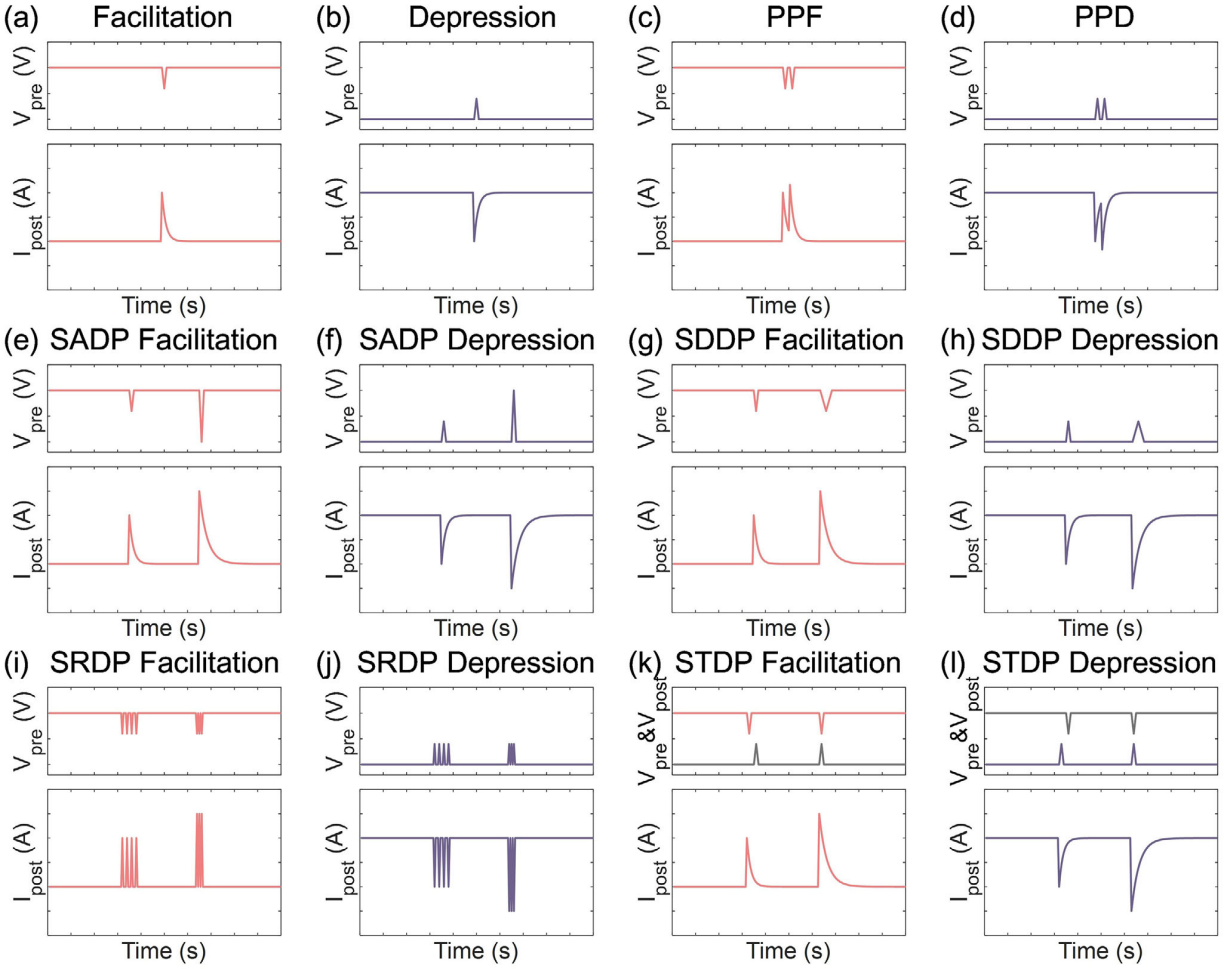
STP characteristics of a typical p‐type depletion‐mode OECS, including a) facilitation, b) depression, c) PPF, d) PPD, e) SADP facilitation, f) SADP depression, g) SDDP facilitation, h) SDDP depression, i) SRDP facilitation, j) SRDP depression, k) STDP facilitation, and l) STDP depression.

STP refers to the dynamic modulation in synaptic functionality on the millisecond‐to‐second timescale in response to stimulation, which mainly reflects the efficiency of neurotransmitter release at presynaptic nerve terminals or the instantaneous adjustment of postsynaptic responses. The two fundamental categories are STP facilitation (**Figure** **5**a) and STP depression (Figure 5b). In biological systems, STP facilitation is typically associated with the prolonged presynaptic calcium ion (Ca^2^⁺) influx, whereas STP depression arises from the rapid depletion of releasable synaptic vesicles from presynaptic terminals or desensitization of postsynaptic receptors. Accordingly, paired‐pulse facilitation (PPF, Figure 5c) and paired‐pulse depression (PPD, Figure 5d) appear as responses to two consecutive pulsed stimuli with enhanced facilitation and depression effects, respectively. Corresponding to OECSs, facilitation refers to an increase in the postsynaptic current (drain current, *I_post_*), while depression results in a current decrease, both induced by the applied presynaptic voltage (gate voltage, *V_pre_*).

The first synaptic demonstration based on OECTs dates back to 2015, when Gkoupidenis et al. constructed a depletion‐mode OECT employing PEDOT:PSS to implement fundamental STP functions, including PPD, adaptation, and dynamic filtering.^[^ ^81^ ^]^ PEDOT:PSS is a p‐type conducting polymer, indicating that the channel exists in a highly conducting state until the introduction of ions, resulting in the de‐doping of the conjugated polymer backbone and the extraction of mobile holes with the help of a positive gate voltage.^[^ ^83^, ^109^ ^]^ When the gate voltage is removed, the injected ions return to the electrolyte, restoring the channel polymer to its original doping state. Due to the slow dynamics of ion migration determining the state retention timescale and intrinsic reversibility of ion injection and extraction, this process is regarded as volatile. Later the same year, they modified the channel material slightly from PEDOT:PSS to poly(3,4‐ethylenedioxythiophene):poly(tetrahydrofuran) (PEDOT:PTHF), introducing conformational rearrangement to realize nonvolatile memory behaviors over several hours achieved by modulating the quantity and spatial interval of input gate voltage pulses.^[^ ^211^ ^]^

Since then, numerous studies have explored versatile device structures and functionalities to replicate biological mechanisms based on the STP in OECSs. For example, several studies employed the vertical structure to fabricate OECSs with high performance.^[^ ^212^, ^213^ ^]^ Additionally, Ji et al. came up with a dual‐gate configuration incorporating a main gate and an auxiliary gate to dynamically modulate the STP characteristics.^[^ ^214^ ^]^ Besides, a dual‐gate configuration can assist in other neuromorphic functionalities, including Boolean logic operations and the emulation of synergistic effects between multiple presynaptic neurons.^[^ ^159^, ^215^ ^]^ Biological neural networks achieve massive interconnectivity through synapses and homeostatic plasticity via global regulation in addition to local regulation. A 4 × 4 array of two‐terminal devices with a global gate voltage and communal electrolyte was developed by Gkoupidenis et al. to investigate the synergistic effect of synaptic strength by local and global stimuli.^[^ ^210^ ^]^ This setup demonstrates that global stimuli can modulate local synaptic strength, with increasing global input suppressing the output of individual synapses. Furthermore, the periodic global input is capable of serving as a clock signal to modulate the transmission of local input pulses, achieving frequency‐selective synchronization.

Beyond the polarity of the applied presynaptic voltage, several additional factors influence synaptic strength, enabling the exploration of advanced plasticity mechanisms for extensive applications. For instance, spike‐amplitude‐dependent plasticity (SADP, Figure 5e–f), spike‐duration‐dependent plasticity (SDDP, Figure 5g,h), spike‐rate‐dependent plasticity (SRDP, Figure 5i,j), and spike‐timing‐dependent plasticity (STDP, Figure 5k,l)^[^ ^216^ ^]^ signify that OECSs perform different responses based on several characteristics of input voltage signals. STDP, in particular, refers to the phenomenon in which the synaptic strength between neurons changes with the timing and interval of the presynaptic and postsynaptic firing spikes, underlying memory encoding and associative learning in biological systems. Harikesh et al. demonstrated STDP by integrating an OECS with a LIF‐OECN based on the A–H model.^[^ ^80^ ^]^ Here, the frequency of output spikes from the OECN is modulated by voltage pulses applied to the OECS, which are then converted into output current pulses according to the synaptic strength of the OECS. According to STDP, in accordance with the Hebbian learning rule, the most substantial change in conductance occurs when presynaptic and postsynaptic spikes are temporally aligned, increasing synaptic strength via a maximal voltage difference for electrical polymerization. This process is akin to the long‐term potentiation process in biological systems, wherein NMDA receptor activation requires coincident presynaptic glutamate release and removal of magnesium ion block by postsynaptic depolarization, effectively functioning as a biological AND gate. Longer overlaps between presynaptic and postsynaptic firing spikes result in larger postsynaptic currents and facilitate OECN spiking frequency, forming a direct mapping relationship between temporal input and output frequency in artificial neural systems.

#### 3.2.2 Long‐Term Plasticity

LTP refers to changes in synaptic connection strength that persist for more than several hours in response to high‐frequency stimulation and involve, for example, gene expression and remodeling of the synaptic structure. In the context of OECSs, LTP potentiation and depression are generally induced by applying a prolonged train of voltage pulses to the presynaptic gate. OECTs operate within electrochemical systems and are vulnerable to self‐discharging as a result of parasitic electrochemical reactions. The intrinsic volatility and instability of conductance modulation in OECTs present significant challenges for the realization of nonvolatile memory functions in neuromorphic computing. Therefore, the development of OECT devices with reliable LTP behaviors, capable of enabling stable long‐term memory and facilitating the transition from STP to LTP, is urgently needed for advancing memory‐mode modulation in artificial neural systems.

**Figure 6:**
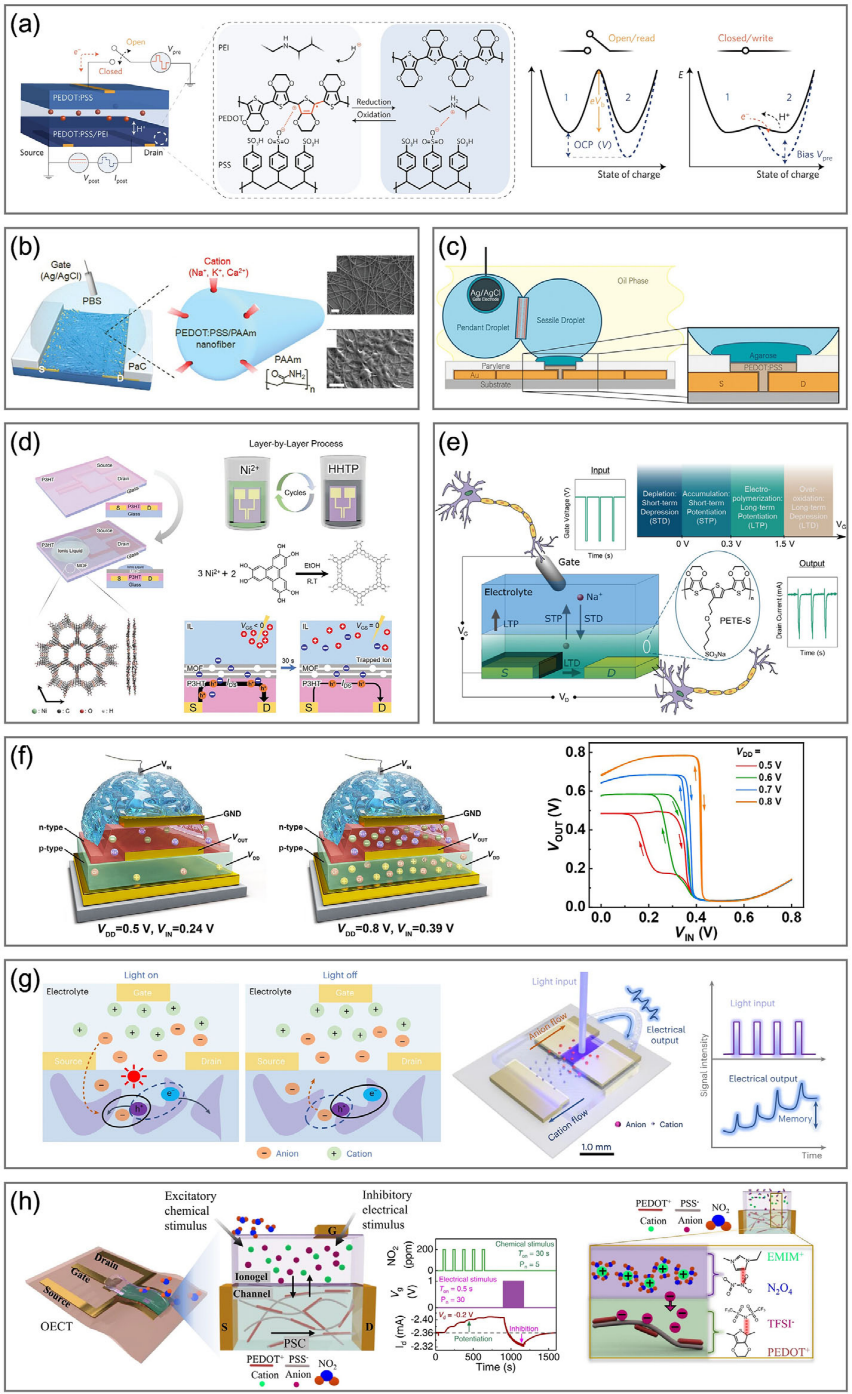
Strategies for achieving LTP in OECSs. a) Nonvolatile OECSs with PEDOT:PSS/PEI as the channel material and PEDOT:PSS as the gate material. Reproduced with permission.^[^ ^164^ ^]^ Copyright 2017, Springer Nature. b) Nonvolatile OECSs with a nanofibrous channel by incorporating PEDOT:PSS and PAAm. Reproduced with permission.^[^ ^173^ ^]^ Copyright 2021, Wiley‐VCH. c) Employing TCDB technology to achieve non‐volatility in OECSs. Reproduced with permission.^[^ ^165^ ^]^ Copyright 2024, Wiley‐VCH. d) Inserting a MOF film to achieve non‐volatility in OECSs. Reproduced with permission.^[^ ^219^ ^]^ Copyright 2024, Wiley‐VCH. e) Evolvable OECSs with PETE‐S as the channel material. Reproduced with permission.^[^ ^78^ ^]^ Copyright 2019, Wiley‐VCH. f) OECS‐based inverters operating in both volatile and nonvolatile modes. Reproduced with permission.^[^ ^130^ ^]^ Copyright 2024, American Association for the Advancement of Science. g). Photon‐modulated nonvolatile OECSs with P3HT:PCBM as the channel material. Reproduced with permission.^[^ ^222^ ^]^ Copyright 2023, Springer Nature. h). Chemically modulated nonvolatile OECSs with a chemically sensitive ion gel as the electrolyte. Reproduced with permission.^[^ ^224^ ^]^ Copyright 2023, Springer Nature.

The interaction between the channel material and electrolyte is a critical determinant of state retention time in OECSs. To extend and stabilize the state retention process for achieving LTP, Burgt et al. developed an architecture with PEDOT:PSS/PEI as the channel material and PEDOT:PSS as the gate material to enable nonvolatile characteristics (**Figure** **6**a).^[^ ^164^ ^]^ Upon the reading (open circuit) condition, the electrolyte acts as an electron‐blocking layer, and the introduction of PEI assists in stabilizing the neutral state of PEDOT through protonation and deprotonation reactions at the amino group, thus steadily maintaining the conductance state in the channel. Besides, the electrostatic potential barrier formed by the difference between the oxidation state and the reduction state of PEDOT is extremely high, ensuring that the state is nonvolatile. During the writing condition, applying a gate voltage can overcome the electrostatic barrier, reduce the energy barrier required for the state transition, and enable conductance regulation at a low voltage. With the help of stable retention and decoupling the writing and reading process, this design supports more than 500 nonvolatile conductance states via a sequence of voltage pulses, offering the possibility for multidigit operations. Another method of incorporating PEDOT:PSS and polyacrylamide (PAAm) with dimethyl sulfoxide (DMSO) treatment to construct a nanofibrous channel was proposed by Lee et al (Figure 6b).^[^ ^173^ ^]^ PAAm effectively forms an ion‐blocking layer and inhibits ion diffusion from the channel to the electrolyte following the channel conductance state transition, resulting in LTP. Furthermore, the 3D structure of nanofibers facilitates omnidirectional ion injection from the electrolyte surface, not only shortening the ion migration distance and enhancing the response speed but also vividly imitating the interconnected architecture of biological neural networks. Furthermore, Kim et al. demonstrated that introducing aminosilane (APT) into the poly(3‐hexylthiophene‐2,5‐diyl) (P3HT) film attains regions of high electron density and effective charge trapping sites due to its amino and silane groups.^[^ ^217^ ^]^ This incorporation expands the hysteresis window of OECSs, suggesting improved charge trapping behavior and enhanced nonvolatile memory performance. In a general way, Lee et al. studied the regulating route for enhancing the doping process and inhibiting the de‐doping process of ions into the channel.^[^ ^218^ ^]^

Inserting an ion‐blocking layer offers an alternative strategy for improving the state retention time in OECSs. For example, Maraj et al. employed transistor‐coupled droplet bilayer (TCDB) technology to form a lipid bilayer between the PEDOT:PSS surface and the suspended Ag/AgCl electrode (Figure 6c).^[^ ^165^ ^]^ This bilayer exhibits high resistance, which can effectively inhibit nonspecific ion diffusion and thereby extend the synaptic state retention time up to 500 s. A similar approach for achieving LTP involves inserting a layer‐by‐layer grown metal–organic framework (MOF) film of Ni‐HHTP between the channel and the electrolyte (Figure 6d).^[^ ^219^ ^]^ The nanoporous structure of the MOF is dimensionally matched to bis(trifluoromethanesulfonyl)imide (TFSI^−^) ions; upon gate voltage application, these ions migrate through the MOF layer and become trapped after the voltage is removed, thus preserving the high conductance state with stable retention.

Gerasimov et al. reported an evolvable OECS based on the p‐type 4‐(2‐(2,5‐bis(2,3‐dihydrothieno[3,4‐b][1,4]dioxin‐5‐yl)thiophen‐3‐yl)ethoxy)butane‐1‐sulfonate (ETE‐S), realizing the transition between STP and LTP (Figure 6e).^[^ ^78^ ^]^ The device operates in a hybrid accumulation‐depletion mode, wherein the drain current can be modulated bidirectionally depending on the magnitude and polarity of the gate voltage. It reveals that low gate voltage pulses induce STP, while constant moderate gate voltage induces LTP because the occurrence of electropolymerization from ETE‐S to PETE‐S depends on the continuous positive excitation of the drain voltage. The drain voltage not only modulates the channel conductance state but also influences the ionic redistribution in the electrolyte. The evolvable OECS represents the first synaptic device capable of self‐generating new synaptic pathways during operation, reminiscent of biological synapse formation and adaptation. Alternatively, Cong et al. proposed an inverter operating in both volatile and nonvolatile modes based on the ambipolar p(gDPP‐V) (Figure 6f).^[^ ^130^ ^]^ Volatility arises under high drain voltages due to ionic equilibrium in the electrolyte, while non‐volatility is achieved at low drain voltages due to ionic imbalance. Another approach to the transition from STP to LTP involves regulating the crystallinity of polymer semiconductor films.^[^ ^198^, ^220^ ^]^ At low gate voltages, ion doping is confined to amorphous domains, leading to volatility, whereas higher voltages enable doping of crystalline domains, resulting in durable and nonvolatile states. Chen et al. developed a novel method for crystallinity manipulation via backbone engineering, offering potential for precise divisional regulation of volatility and non‐volatility.^[^ ^221^ ^]^

Novelly, inspired by the visual system, Chen et al. constructed a photon‐modulated OECS via combining P3HT and [6,6]‐phenyl‐C 61‐butyric acid methyl ester (PCBM) as the channel material (Figure 6g).^[^ ^222^ ^]^ The bulk heterojunction of P3HT and PCBM generates electronic carriers when receiving light, which forces the ions in the electrolyte to dope into the channel. Once the light is removed, the presence of ions inhibits charge recombination and forms a nonvolatile current, enabling channel conductance modulation for optical writing and electrical erasure. Additionally, the current response is modulated by the light intensity and wavelength, allowing for the discrimination of different optical signals and mimicking the brightness and color perception of human vision. In another means, Liu et al. combined P3HT and chlorophyll as the channel material.^[^ ^223^ ^]^ The EDL formed by the injected anions and holes at the heterojunction when the light turns on reduces the recombination of conductive holes and thus facilitates nonvolatile memory. Likewise, drawing inspiration from the olfactory system, Chouhdry et al. employed a chemically sensitive ion gel as the electrolyte to realize chemically modulated OECSs with nonvolatile memory (Figure 6h).^[^ ^224^ ^]^ Given that nitrogen dioxide (NO_2_) forms stable composites with the cations in the electrolyte, the regulated state could endure for a considerable amount of time, providing the basis for chemical stimulation and electrical inhibition.

Beyond modifying the channel material and inserting an ion‐blocking layer between the channel and the electrolyte, gate engineering could also provide a promising strategy for harvesting nonvolatile characteristics. For instance, Battistoni et al. employed multilayer graphene as the gate material to achieve more than 100 nonvolatile memory states through a significant hysteresis effect.^[^ ^166^ ^]^ The nonuniform structure of multilayer graphene consists of stacks of polygonal sheets containing abundant defects and disordered sites that provide effective adsorption sites for ions, supporting the stable retention of oppositely charged ions in the channel, which forms a nonvolatile resistive state due to the binding of ions by the multilayer graphene gate.

### 3.3 Integrated Devices

Biological neurons convert electrical signals into chemical signals (release of neurotransmitters from the presynaptic membrane) through synapses, and then into electrical signals (changes in postsynaptic membrane potential), thereby realizing information transmission in the neural network, wherein the architecture integrated by an OECN and an OECS forms the basic building block of their artificial counterparts.

**Figure 7:**
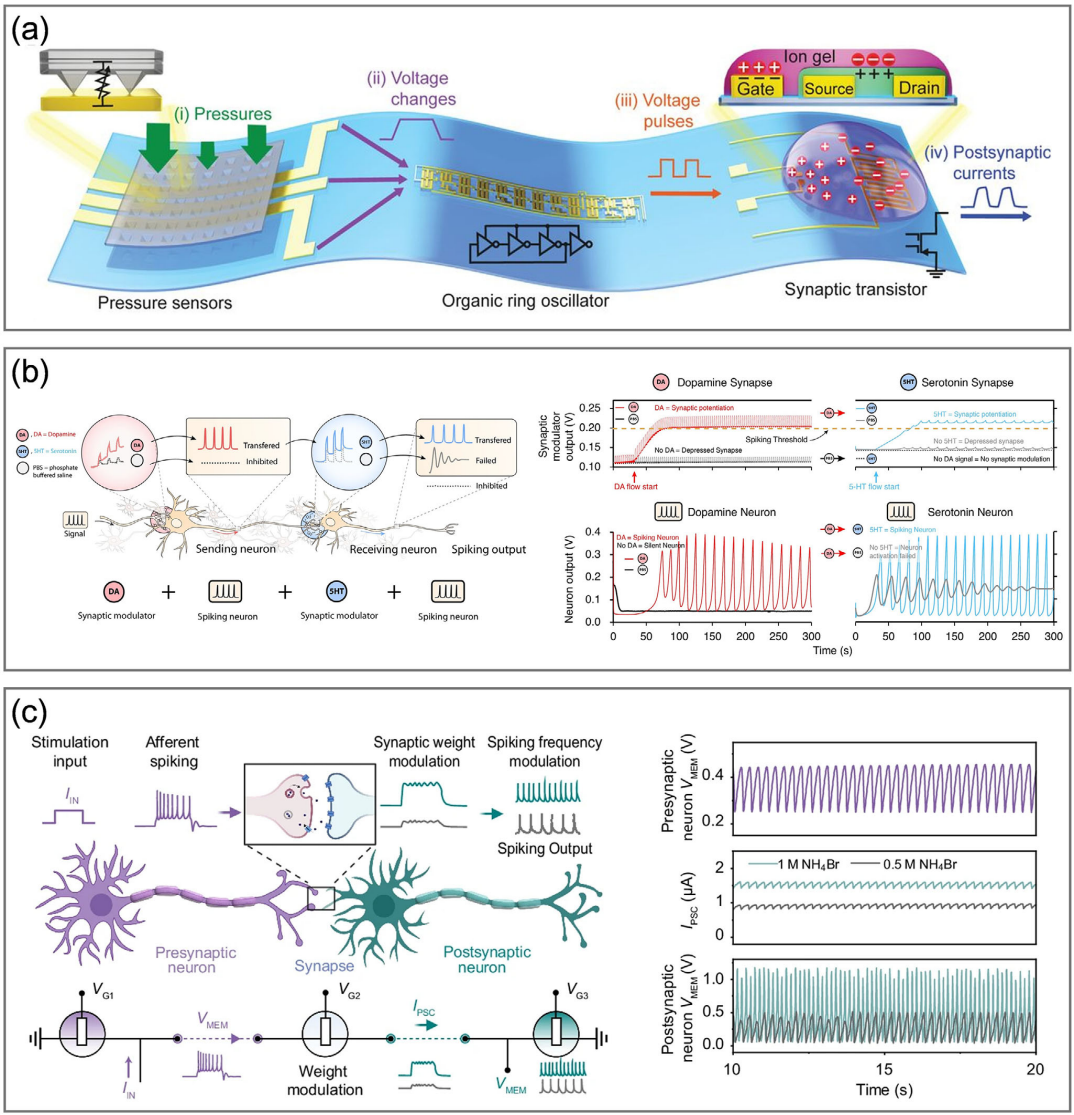
Integrated devices of OECSs and OECNs. a) Flexible organic artificial afferent nerves by integrating three main parts: a resistive pressure sensor as the receptor, an organic ring oscillator as the artificial neuron, and an EGOFETs as the artificial synapse. Reproduced with permission.^[^ ^13^ ^]^ Copyright 2018, American Association for the Advancement of Science. b) Organic artificial nerves for dopamine and serotonin detection by cascading multiple OECNs and OECSs. Reproduced with permission.^[^ ^157^ ^]^ Copyright 2024, Springer Nature. c) Compact artificial presynaptic neuron−synapse−postsynaptic neuron nerves by cascading three individual OECTs. Reproduced with permission.^[^ ^72^ ^]^ Copyright 2025, Springer Nature.

In 2018, Kim et al. introduced a flexible organic artificial afferent neural system that emulated the behaviors of mammalian slowly adapting type I (SA‐I) sensory neurons, inspired by the distributed information processing of biological somatosensory nerves (**Figure** **7**a).^[^ ^13^ ^]^ The system combines tactile sensing, spike generation, and synaptic signal processing to emulate realistic neural operations by integrating three main parts: a resistive pressure sensor as the receptor, an organic ring oscillator as the artificial neuron, and an electrolyte‐gated OFET (EGOFET) as the artificial synapse. The ring oscillator establishes a real‐time one‐to‐one relationship, which generates an oscillating signal with fixed frequency and amplitude in response to a constant voltage input from the pressure sensor with a fixed pressure. Notably, both the frequency and peak amplitude of the output spikes increase with increasing applied pressure. Besides, to emulate the parallel processing of SA‐I afferent neurons forming synapses with multiple interneurons in the spinal cord, manufactured from a p‐type conjugated polymer with solution treatment and an ion gel, the EGOFET may integrate electrical signals from multiple ring oscillators.

By replacing the ring oscillator with a LIF‐OECN based on the A−H model, Matrone et al. developed an artificial nerve through the integration of a physical receptor, an OECS, and two OECNs (Figure 7b).^[^ ^157^ ^]^ In this system, the light sensor transduces light intensity into receptor potential, which is subsequently input into the OECN. Upon reaching the threshold voltage, the OECN discharges and generates an output spiking signal whose frequency varies with light intensity, which is then fed into the OECS to modulate the synaptic weight accordingly. In the presence of neurotransmitters (e.g., dopamine, serotonin), spiking signals act on the synaptic gate, triggering an oxidative reaction that results in nonvolatile modulation of synaptic strength and affecting the spiking frequency of the postsynaptic neuron. To emulate the retinal preprocessing functionalities, light stimulus is sequentially processed through dopamine‐regulated synapses before reaching serotonin‐modulated neurons. This hierarchical arrangement is realized by cascading multiple OECNs and OECSs, thereby converting light intensity into dynamic variations in spiking frequency and synaptic weight.

Thanks to the compendious structure of 1T‐OECNs, a compact artificial presynaptic neuron−synapse−postsynaptic neuron neural system has been constructed using only three individual OECTs (Figure 7c).^[^ ^72^ ^]^ Modulating the electrolyte concentration at the synapse precisely tunes the postsynaptic current and, consequently, the output spiking frequency of the postsynaptic neuron, thereby effectively reproducing the weight−frequency correlation of biological synapses. The integrated system successfully emulates neural signal transmission and STDP. Additionally, a completely functional artificial afferent nerve system can be established by interfacing with pressure sensors, enabling the transduction of mechanical stimuli into neuromorphic pulse trains.

## 4 Applications of OECT‐Based Neuromorphic Devices

### 4.1 Boolean Logic Operations

Boolean logic operations are based on binary values and involve evaluating the relationships between propositions using logical operators. They serve as the theoretical cornerstone of modern computer science, digital circuit design, and logical reasoning. In integrated circuits, Boolean logic operations are widely employed for computation and control. Recent advancements in OECSs and OECNs have demonstrated their potential to perform various types of Boolean logic operations.^[^ ^153^, ^184^, ^244^, ^245^, ^246^, ^247^ ^]^

**Figure 8:**
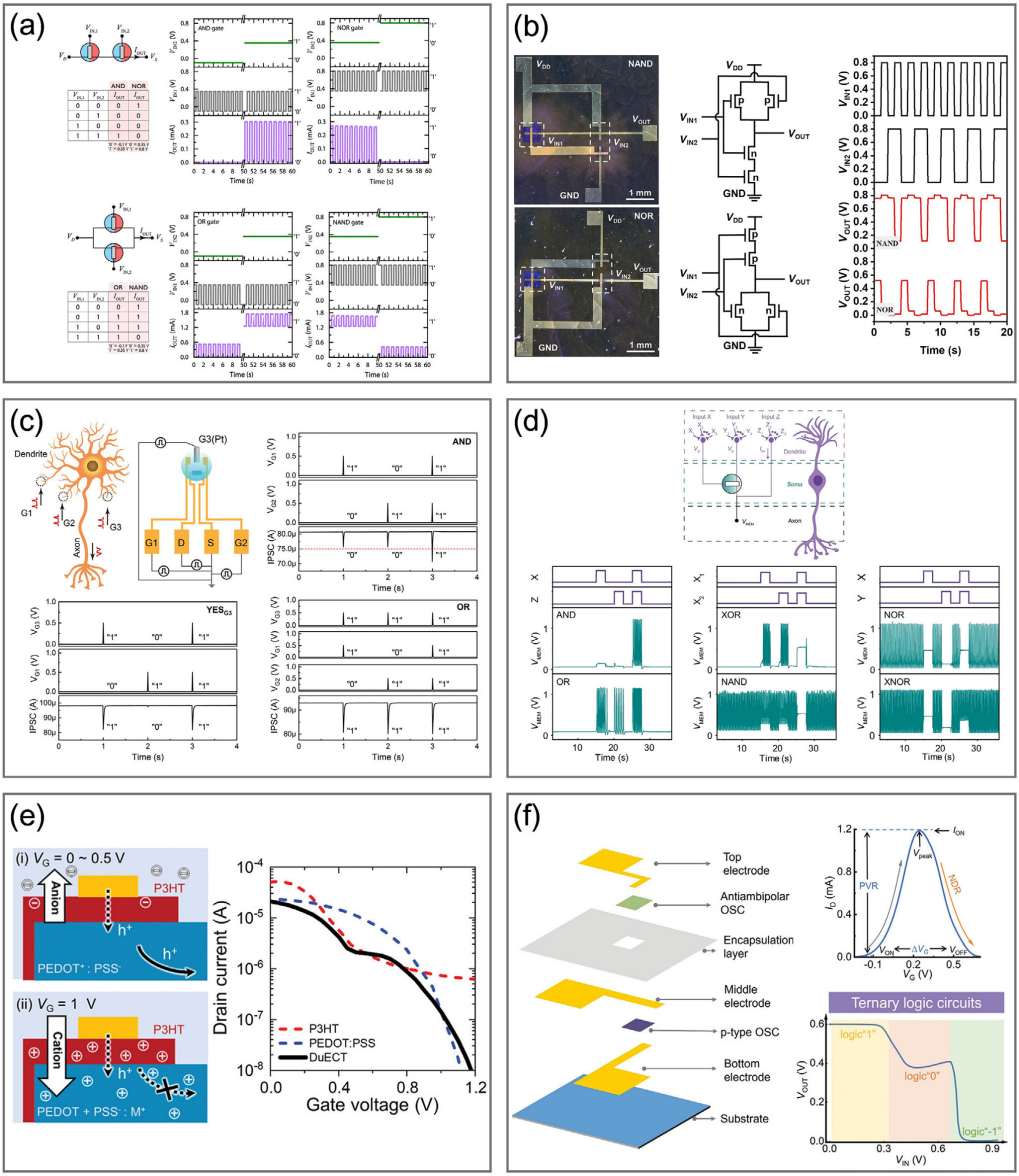
Boolean logic operations based on OECSs and OECNs. a) Operations of AND, OR, NAND, and NOR achieved by two bilayer OECSs in series or parallel. Reproduced with permission.^[^ ^178^ ^]^ Copyright 2024, Springer Nature. b) Operations of NAND and NOR achieved by two p‐type OECSs and two n‐type OECSs. Reproduced with permission.^[^ ^130^ ^]^ Copyright 2024, American Association for the Advancement of Science. c) Operations of AND, OR, and YES (selectivity) achieved by a single OECS with three gate electrodes. Reproduced with permission.^[^ ^202^ ^]^ Copyright 2019, Wiley‐VCH. d) Operations of AND, OR, NAND, NOR, XOR, and XNOR achieved by a 1T‐OECN. Reproduced with permission.^[^ ^72^ ^]^ Copyright 2025, Springer Nature. e) Ternary logic achieved by a tandem structure of two p‐type depletion‐mode channels. Reproduced with permission.^[^ ^248^ ^]^ Copyright 2023, Wiley‐VCH. f) Ternary logic achieved by a tandem structure of an antiambipolar channel and a p‐type channel. Reproduced with permission.^[^ ^249^ ^]^ Copyright 2024, Wiley‐VCH.

By leveraging the switching characteristics of OECSs, basic Boolean logic operations such as AND, OR, NAND, and NOR can be readily implemented by connecting two OECSs in series or parallel configurations (**Figure** **8**a).^[^ ^199^ ^]^ In another way, Cong et al. fabricated conventional Boolean logic circuits of NAND and NOR based on the volatile mode of ambipolar p(gDPP‐V), each comprising two OECSs operating in p‐type mode and two OECSs operating in n‐type mode, respectively (Figure 8b).^[^ ^130^ ^]^ Another alternative method composed of multiple gate electrodes as inputs in a single OECS was employed for Boolean logic operations, including AND, OR, and YES (selectivity) by Liu et al (Figure 8c).^[^ ^202^ ^]^ By exploiting the spatial capacitive coupling characteristics among three gate electrodes, the electric fields generated by multiple input terminals can be superimposed to modulate the channel conductance, thereby achieving the integration and logical operations of input signals based on the predetermined threshold value of logic. Such Boolean logic operations emulate the dendritic integration observed in biological neurons, offering a biomimetic strategy for constructing neuromorphic chips with spatiotemporal information processing capability.

Recently, Ji et al proposed a significantly simplified implementation of Boolean logic operations by 1T‐OECNs (Figure 8d).^[^ ^72^ ^]^ Different from the constant output of OECS‐based Boolean logic circuits, the output of OECN‐based Boolean logic circuits consists of alternating voltage spikes. The channel, drain, gate, and source electrodes of the 1T‐OECN correspond to the soma, axon, and dendrite of biological neurons, respectively. Through treating the three terminals (gate, source, drain) as inputs, the drain voltage as output, and leveraging the nonvolatile hysteresis effect, several Boolean logic operations are available, including AND, OR, NAND, NOR, XOR, and XNOR. While conventional Boolean logic circuits require multiple transistors or passive components, however, such 1T‐OECN enables multiple logic operations through a single device, thereby dramatically simplifying circuit complexity.

Beyond the binary value employed in conventional Boolean logic operations, multi‐value logic represents a pivotal advancement in the supramolar era, where a single device can encode multiple logic states, thereby increasing information density and reducing circuit complexity and power consumption. Lim et al. proposed a tandem vertical structure of OECS based on P3HT and PEDOT:PSS to achieve ternary logic (Figure 8e).^[^ ^248^ ^]^ The two p‐type depletion‐mode channels form a tandem configuration with distinct off voltages: both channels are on‐state without gate voltage; P3HT channel turns off, and PEDOT:PSS remains on‐state under a low gate voltage; both channels are off‐state under a high gate voltage. The three conductance states offer the basis for ternary logic operations by a single device, with the conductance of the intermediate state modulated by the P3HT film thickness. Likewise, another tandem OECS composed of the antiambipolar small molecule t‐gdiPDI and p‐type poly[3,3′‐bis[2‐[2‐(2‐methoxyethoxy)ethoxy]ethoxy]‐2,2′:5′,2″‐terthiophene‐5,5″‐diyl] (p(g2T‐T)) was reported by Deng et al (Figure 8f).^[^ ^249^ ^]^ In the configuration, the output voltage logic is determined by the resistance ratio of the antiambipolar channel and p‐type channel. Additionally, the vertical structure not only provides the foundation for the construction of ternary logic but also improves the performance of OECSs and the speed of Boolean logic operations.

### 4.2 Reservoir Computing

RC represents a novel type of recurrent neural network (RNN) framework, in which time‐series data are processed through a randomly generated dynamic system known as the reservoir pool. The output weights are solved via a single linear regression, eliminating the need for backpropagation, demonstrating high computational efficiency and powerful performance in large‐scale time‐series prediction and pattern recognition tasks. Thanks to the nonlinear tunability, STP, and LTP of OECSs, they are emerging as exceptional candidates for implementing RC neural networks and their applications, highlighting the biomimetic stochasticity and global interconnectivity observed in the human brain.^[^ ^250^, ^251^, ^252^, ^253^ ^]^

**Figure 9:**
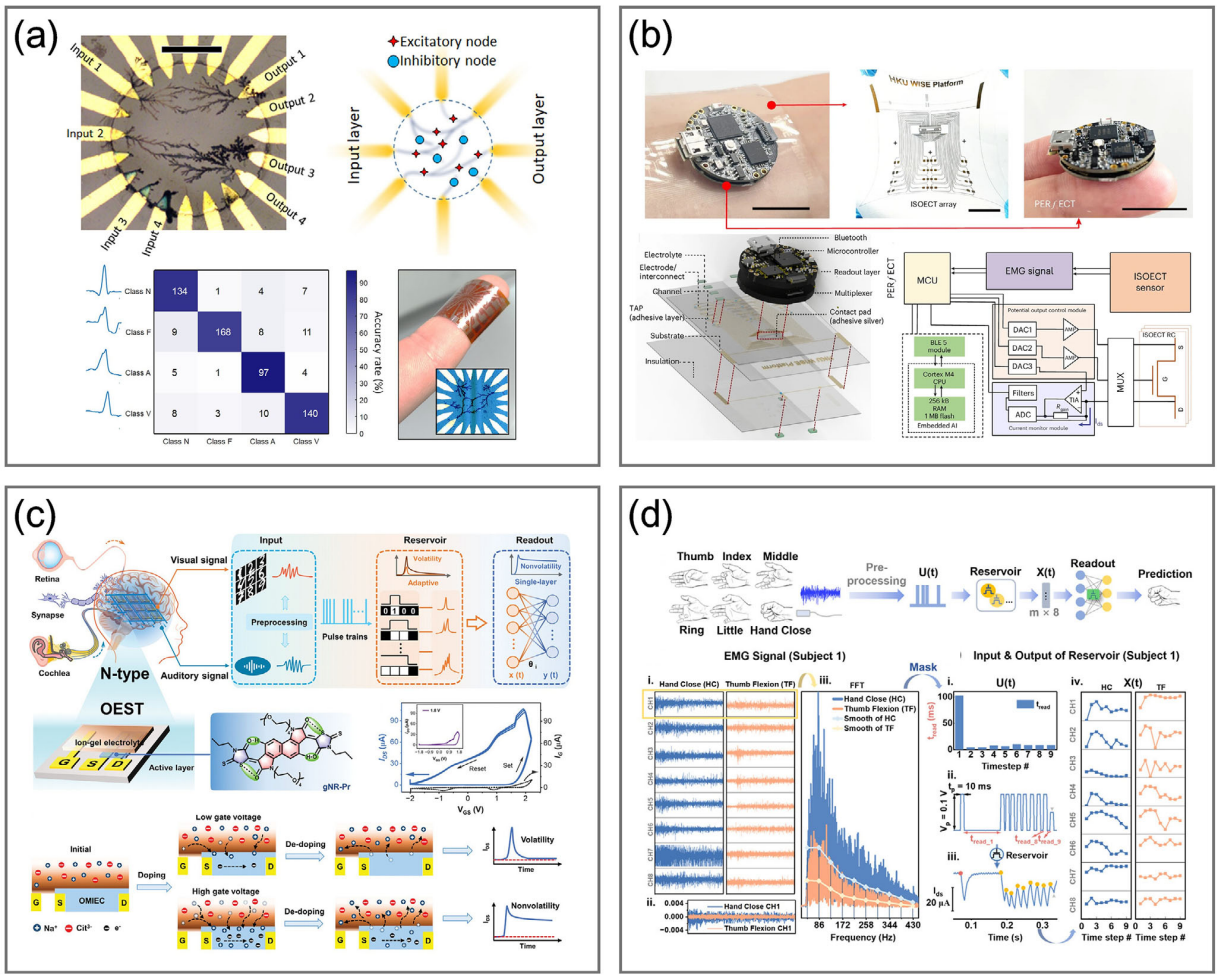
RC neural networks based on OECSs. a) The first OECS‐based RC neural network constructed by multiple fibrous channels. Reproduced with permission.^[^ ^227^ ^]^ Copyright 2021, American Association for the Advancement of Science. b) RC neural networks constructed by an intrinsically stretchable OECS array and a peripheral coin‐sized readout circuit for MNIST dataset classification and gesture recognition.^[^ ^254^ ^]^ Copyright 2024, Springer Nature. c) RC neural networks based on the tunability between volatility and non‐volatility of n‐type material gNR‐Pr. Reproduced with permission.^[^ ^237^ ^]^ Copyright 2025, Wiley‐VCH. d). RC neural networks for EMG signal classification.^[^ ^239^ ^]^ Copyright 2025, Wiley‐VCH.

In 2021, Cucchi et al. proposed the first RC implementation utilizing fibrous OECSs based on poly(3,4‐ethylenedioxythiophene):hexafluorophosphate (PEDOT:PF_6_) (**Figure** **9**a).^[^ ^227^ ^]^ Benefiting from the balanced intra‐ and inter‐coupling of the electrons and ions within the multiple fibrous OMIEC channels, the fibrous network achieves nonlinear mapping from input voltage signals to output voltage signals for further feature extraction. The accumulation of ions at the fiberadvs73345electrolyte interface is time‐dependent, rendering the network state not only dependent on current inputs but also influenced by recent inputs, which form the short‐term memory for processing temporal signals (e.g., heartbeat signals). Furthermore, the random AC‐electropolymerization of PEDOT:PF_6_ fibers fulfills the stochasticity of the reservoir pool, endowing diverse dynamic characteristics for signal processing and eliminating the need for precise patterning.

Liu et al. developed an intrinsically stretchable OECS array integrated with a coin‐sized readout circuit for realistic tasks based on the RC neural network (Figure 9b).^[^ ^254^ ^]^ The nonlinear features of OECSs are well‐suited to the operational requirements of RC, as their gate electrodes can detect electrochemical signals and nonlinearly modulate ion transport and electrochemical doping and de‐doping processes within the channel. Each unit in the 4 × 4 array serves as a dynamic node within the reservoir pool, enabling the processing of four‐bit pulse inputs into 16 distinguishable output current patterns. When combined with a coin‐sized readout system, signal acquisition, amplification, and RC output processing are integrated to establish a complete sensing‐computing closed loop for applications such as MNIST dataset classification and gesture recognition. Additionally, this RC neural network only trains the sparse connection weights between the reservoir pool and the output layer, rather than the entire network, thereby significantly reducing both the operating voltage to 4 mV and power consumption to 36 nW.

Lately, Liu et al. reported a RC neural network based on the high‐performance n‐type material gNR‐Pr (Figure 9c).^[^ ^237^ ^]^ Different from the aforementioned system incorporated a peripheral readout circuit, this ANN is entirely composed of OECSs. Due to their ability to transition from STP to LTP, modulated by gate voltage and pulse duration, STP is suitable for the volatile memory requirements of the reservoir pool, while LTP fulfills the nonvolatile storage needs of the readout layer, respectively. In the reservoir pool construction, temporal input signals are mapped into a high‐dimensional space through volatile dynamics, facilitating nonlinear feature extraction. In contrast, non‐volatility in the readout layer enables linear classification and stable weight retention. The RC neural network has demonstrated successful application in tasks such as image and snoring recognition. Notably, the energy consumption per spike of a single OECS can be as low as 47 fJ, approaching the efficiency of biological systems, rendering it suitable for wearable or real‐time computing scenarios. A similar RC architecture based on PEDOT:Tos/PTHF employs both the volatile and nonvolatile characteristics to train and predict extra electromyography (EMG) signals (Figure 9d).^[^ ^239^ ^]^ EMG signals are converted into the frequency domain via fast Fourier transform (FFT), and the spectral features are mapped onto pulse parameters using mathematical masks, which are then fed into the reservoir pool composed of STM‐mode OECSs. Following extraction, the readout layer, made up of LTM‐mode OECSs performs the state decoding. Such RC neural network has been employed in various physiological signal detection, including speech,^[^ ^241^ ^]^ EMG,^[^ ^240^ ^]^ and electrocardiogram (ECG) signals.^[^ ^255^ ^]^

### 4.3 Pavlovian Conditioning (Classical Conditioning)

**Figure 10:**
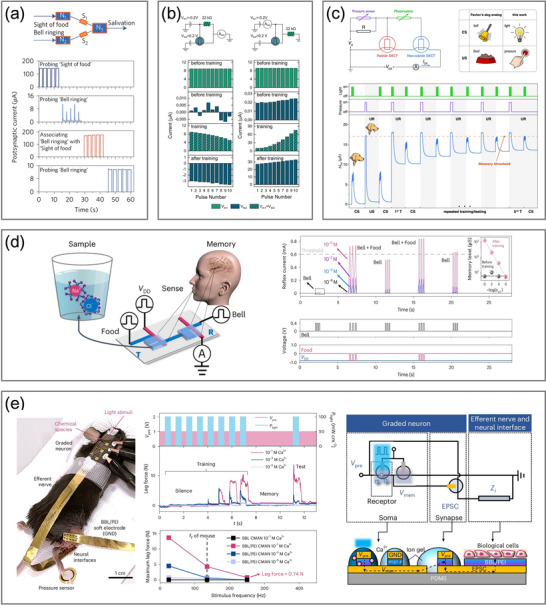
Pavlovian conditioning validation based on OECSs and OECNs. a) Associative learning of the bell and food by OECSs. Reproduced with permission.^[^ ^164^ ^]^ Copyright 2017, Springer Nature. b) Destructive and constructive conditioning models by detecting the output current at different positions. Reproduced with permission.^[^ ^78^ ^]^ Copyright 2019, Wiley‐VCH. c) Integration of volatile and nonvolatile OECSs with a pressure sensor and a photoresistor, respectively, for associative learning of the light and pressure.^[^ ^228^ ^]^ Copyright 2021, Springer Nature. d) 1T1R configurations of OECSs for associative learning of the bell and food. Reproduced with permission.^[^ ^233^ ^]^ Copyright 2023, Springer Nature. e) Biological level associative learning based on the synergistic effect of the light and chemically modulating calcium ions to trigger the leg of a mouse by artificial nerves consisting of LIF‐OECNs. Reproduced with permission.^[^ ^196^ ^]^ Copyright 2025, Springer Nature.

Pavlovian conditioning (classical conditioning) reveals the basic principle of learning, i.e., stimulus association, which not only lays the foundation for behaviorist psychology but also provides a core framework for understanding learning and memory mechanisms in the brain (e.g., synaptic plasticity). Burgt et al. validated Pavlovian conditioning by OECSs for the first time (**Figure** **10**a).^[^ ^164^ ^]^ In the circuit simulating Pavlovian conditioning, STDP is adopted to establish an association between the pulse timing of a bell and the sight of food. When the two stimulating pulses appear in close temporal succession, the weight of the artificial synapse is enhanced according to the STDP rule, thereby inducing a conditioned salivation response to the bell alone and achieving associative learning and memory. Later, by detecting the output current at different positions, destructive and constructive Pavlovian conditioning models based on the electropolymerized material PETE‐S are established to investigate the mechanisms underlying adversarial and collaborative learning, respectively (Figure 10b).^[^ ^78^ ^]^

Another circuit, composed of a photoresistor and a pressure sensor, is constructed to further explore the integration of OECSs with physical sensors in associative learning systems (Figure 10c).^[^ ^228^ ^]^ In this case, memory storage employs the nonvolatile OECS, while short‐term signal processing employs the volatile OECS. The formation of associative learning requires simultaneous application of both stimuli, with voltage pulses operating on both volatile and nonvolatile OECSs over multiple iterations. A one‐transistor and one‐resistor (1T1R) configuration is also employed to realize classical conditioning (Figure 10d).^[^ ^233^ ^]^ By leveraging volatile and non‐volatile characteristics under different applied gate voltages, an association between the bell and food pulses is formed following repeated simultaneous training cycles.

More recently, in contrast to prior simulation‐level research, Wang et al. experimentally validated classical conditioning in a biological context using a LIF‐OECN (Figure 10e).^[^ ^196^ ^]^ By simultaneously applying light stimulation and chemically modulating calcium ions, the synergistic effect of the two factors enhances the synaptic strength of the nerve, enabling the learning transition from no response to a specific response to the light stimulus. During this learning process, the light stimulus serves as the triggering signal, while calcium ion concentration acts as a modulating factor, closely resembling the conditioning mechanism of biological neurons' electrical signal triggering and calcium ion regulation. Remarkably, even when exposed to light pulses at 250 Hz, which exceeds the critical firing frequency of mouse neurons (135 Hz), classical conditioning is still achieved, demonstrating behavioral learning beyond the typical biological frequency limit.

### 4.4 Pattern Recognition

Pattern recognition is a fundamental field within AI and computer science, focusing on enabling machines to identify, classify, and interpret patterns across diverse data modalities. Its primary objective is to replicate the cognitive functions of the human brain so that AI may derive significant insights and conduct data‐driven decisions, which is generally achieved by ANNs. To date, several implementations of pattern recognition based on OECSs and OECNs have been demonstrated.^[^ ^157^, ^256^ ^]^

**Figure 11:**
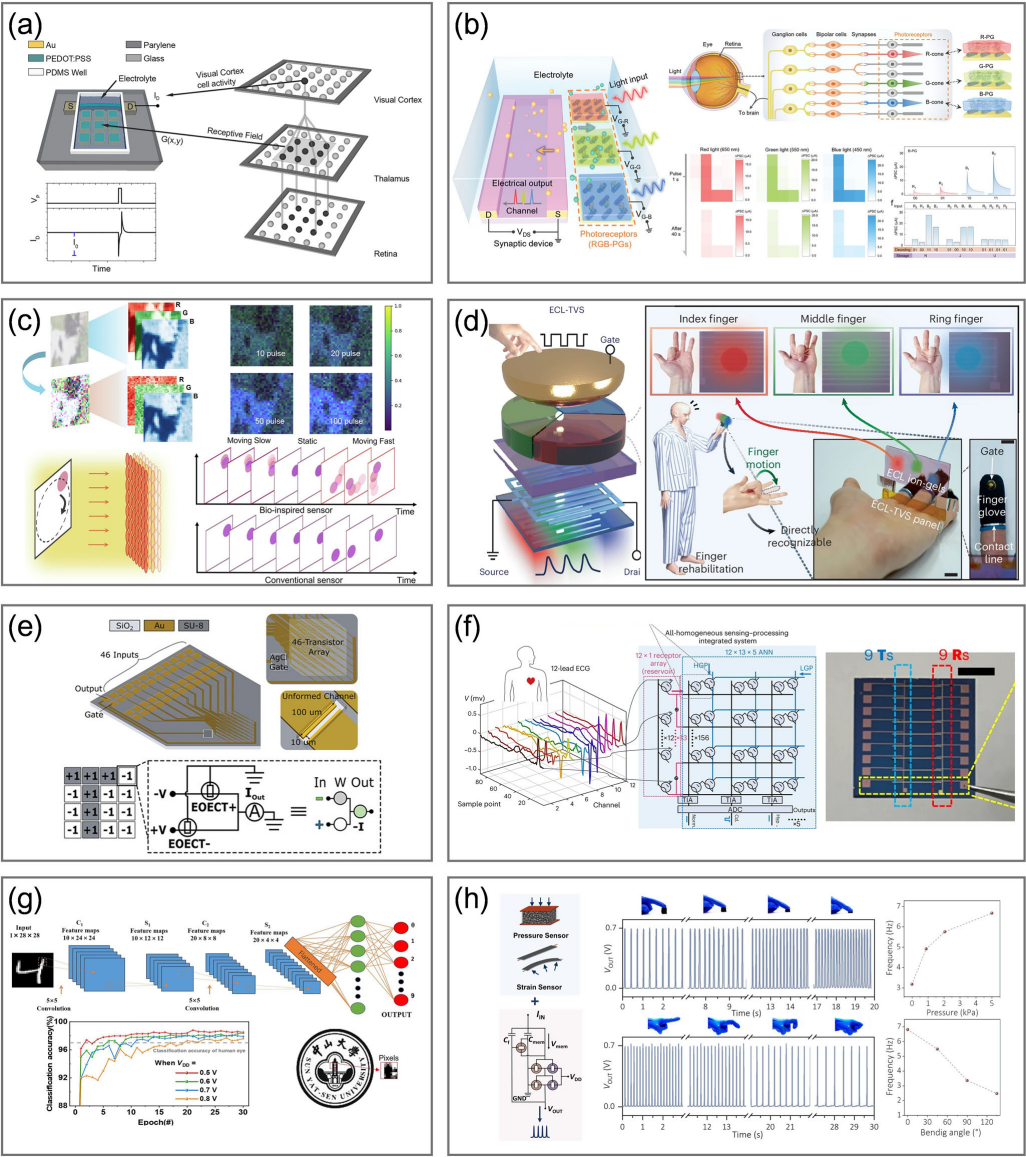
Pattern recognition tasks based on OECSs and OECNs. a) Spatiotemporal processing by multi‐gate OECSs. Reproduced with permission.^[^ ^201^ ^]^ Copyright 2016, Springer Nature. b) RGB component recognition of an “L” pattern by 4 × 4 retinomorphic OECS arrays. Reproduced with permission.^[^ ^234^ ^]^ Copyright 2024, Wiley‐VCH. c). Selective augmentation of particular color channels and motion detection by OECS arrays. Reproduced with permission.^[^ ^242^ ^]^ Copyright 2025, Springer Nature. d). Visualization in the finger rehabilitation task by OECSs. Reproduced with permission.^[^ ^258^ ^]^ Copyright 2025, Springer Nature. e) Image pattern classification of ‘T’ and ‘J’ on the touchpad by OECS arrays with bidirectional modulation. Reproduced with permission.^[^ ^79^ ^]^ Copyright 2023, Wiley‐VCH. f) Classifying five categories of 12‐lead ECG data by OECS‐based ANNs. Reproduced with permission.^[^ ^233^ ^]^ Copyright 2023, Springer Nature. g) Image pattern classification by the integration of OECS arrays and CNNs. Reproduced with permission.^[^ ^130^ ^]^ Copyright 2024, American Association for the Advancement of Science. h) Pressure and image pattern classification by SNNs based on LIF‐OECNs. Reproduced with permission.^[^ ^131^ ^]^ Copyright 2025, National Academy of Sciences.

Inspired by the directional selectivity observed in the visual cortex, a typical spatiotemporal processing function, Gkoupidenis et al. constructed a gate array to emulate the retinal receptive field and analogized the drain current to the discharging activity of visual cortical neurons, realizing the mapping of the spatial input voltage pattern (vector field) to a single current output (scalar) based on such multi‐gate OECSs (**Figure** **11**a).^[^ ^201^ ^]^ Traditional direction‐selective devices (e.g., memristor grids and floating‐gate transistor arrays) rely on resistance or current mapping of multiple device arrays. However, this design demonstrates spatiotemporal processing through a single multi‐gate OECS, thereby simplifying hardware complexity. A similar directional selectivity is achieved by detecting the magnitude of response spikes based on diketopyrrolopyrrole thieno[3,2‐b]thiophene copolymer (DPPT‐TT) as the channel material and chitosan as the electrolyte.^[^ ^236^ ^]^ Drawing inspiration from the color perception of cone cells in the retina, Hu et al. fabricated a multicolor hydrogel‐based photoelectrochemical retinomorphic synapse array comprising red, green, and blue OECSs (Figure 11b).^[^ ^234^ ^]^ By modulating the ion transport using photostimulation instead of applied gate voltages, the devices enable significant energy reduction and compatibility with biological integration. A 4 × 4 array successfully recognizes the RGB components of an “L” pattern and retains memory for 40 s after the light is switched out. Likewise, Wang et al. expanded the light absorption range to UV–VIS–NIR by exploiting the single‐component n‐type p(C2F‐z) (Figure 11c).^[^ ^242^ ^]^ By utilizing the wavelength‐specific photoresponse of p(C2F‐z), the OECS allows for the selective augmentation and noise reduction of particular color channels in a colorful image without the need for intricate external circuits. Additionally, A 32 × 32 OECS array is constructed to extract spatial and temporal information of moving objects simultaneously to realize motion detection thanks to the spatial difference of the array and the time‐cumulative effect of a single OECS. Likewise, Xu et al.^[^ ^257^ ^]^ and Xiang et al.^[^ ^243^ ^]^ reported such devices based on P3HT and P3HT:Y6 as the channel material, respectively. Different from light perception, Kim et al. proposed an OECS integrating tactile sensing and luminescence in a single device inspired by the tactile‐luminescent synergistic mechanisms in sea snails (Figure 11d).^[^ ^258^ ^]^ Through the transition from pressure signals to optical signals, the devices successfully achieve visualization in the finger rehabilitation task.

Gerasimov et al. proposed a pattern classifier based on evolvable OECSs with PETE‐S as its channel material (Figure 11e).^[^ ^79^ ^]^ By constructing a 4 × 4 pixel array and employing the Widrow–Hoff algorithm, the ANN classifies the image patterns of “T” and “J” on the touchpad accurately and effectively. While typical OECS‐based ANNs support only unidirectional synaptic weight changes via electropolymerization, this design splits each pixel weight into two components, i.e., positive and negative, and represents the synaptic weight by the conductance difference, achieving bidirectional modulation. Additionally, by connecting this ANN to the ventral nerve cord of the leech, pattern recognition‐driven biological responses have been successfully achieved. In another study, Wang et al. reported a 12 × 13 × 5 ANN for classifying five categories of 12‐lead ECG data (Figure 11f).^[^ ^233^ ^]^ RC is employed between the receptor and ANN layers to perform dimensionality reduction. In simulated diagnosis, the device model based on experimental calibration achieves a diagnostic accuracy of 100% after 700 training epochs. A novel architecture is developed by integrating an array of inverters fabricated with ambipolar OECSs with a convolutional neural network (CNN) (Figure 11g).^[^ ^130^ ^]^ Nonvolatile voltage outputs are treated as fixed weights, allowing the weight matrix in the CNN to be retrieved from the voltage transfer characteristics of the inverter upon MNIST image encoding. The system reaches a classification accuracy of 98.5% after 15 training epochs under a low working voltage of 0.5 V for MNIST pattern classification tasks. Moreover, the image memory duration can be tuned by adjusting the magnitude of drain voltage, enabling the inverter to function in either volatile or nonvolatile mode, thus replicating the short‐term and long‐term pattern memory in the human neural system.

OECNs could also be employed in pattern recognition applications. Yao et al. developed a pressure‐sensitive network based on high‐performance LIF‐OECNs (Figure 11h).^[^ ^131^ ^]^ The resistance of carbon nanotube (CNT) foam decreases under pressure, which increases the input current of OECN through the voltage divider circuit and enhances the output frequency. Conversely, the resistance of CNT‐coated rubber gloves increases when bent, which decreases the input current, and the output frequency shrinks as the bending angle increases. An input layer consisting of 784 OECNs receives the pressure signals from each pixel of the handwritten digit in the MNIST dataset and is trained by a three‐layer SNN (784 × 196 × 10) to optimize the synaptic weights via the backpropagation algorithm. After 10,000 training epochs, this artificial SNN achieves a recognition accuracy of 96.16%, and the confusion matrix demonstrates the preponderance of diagonal elements, suggesting effective pressure distribution recognition of images.

### 4.5 Biointerfaces

A biointerface is defined as a boundary where biological systems (e.g., cells, tissues, organisms) interact with electronic devices. It enables bidirectional communication, allowing biological signals (e.g., physical, chemical, and electrical) to be transduced into electrical forms and vice versa. For the effective and scalable integration of biological systems with OECT‐based neuromorphic devices, biointerfaces should exhibit excellent biocompatibility and long‐term stability to ensure biorealistic functionalities.^[^ ^73^, ^259^ ^]^

**Figure 12:**
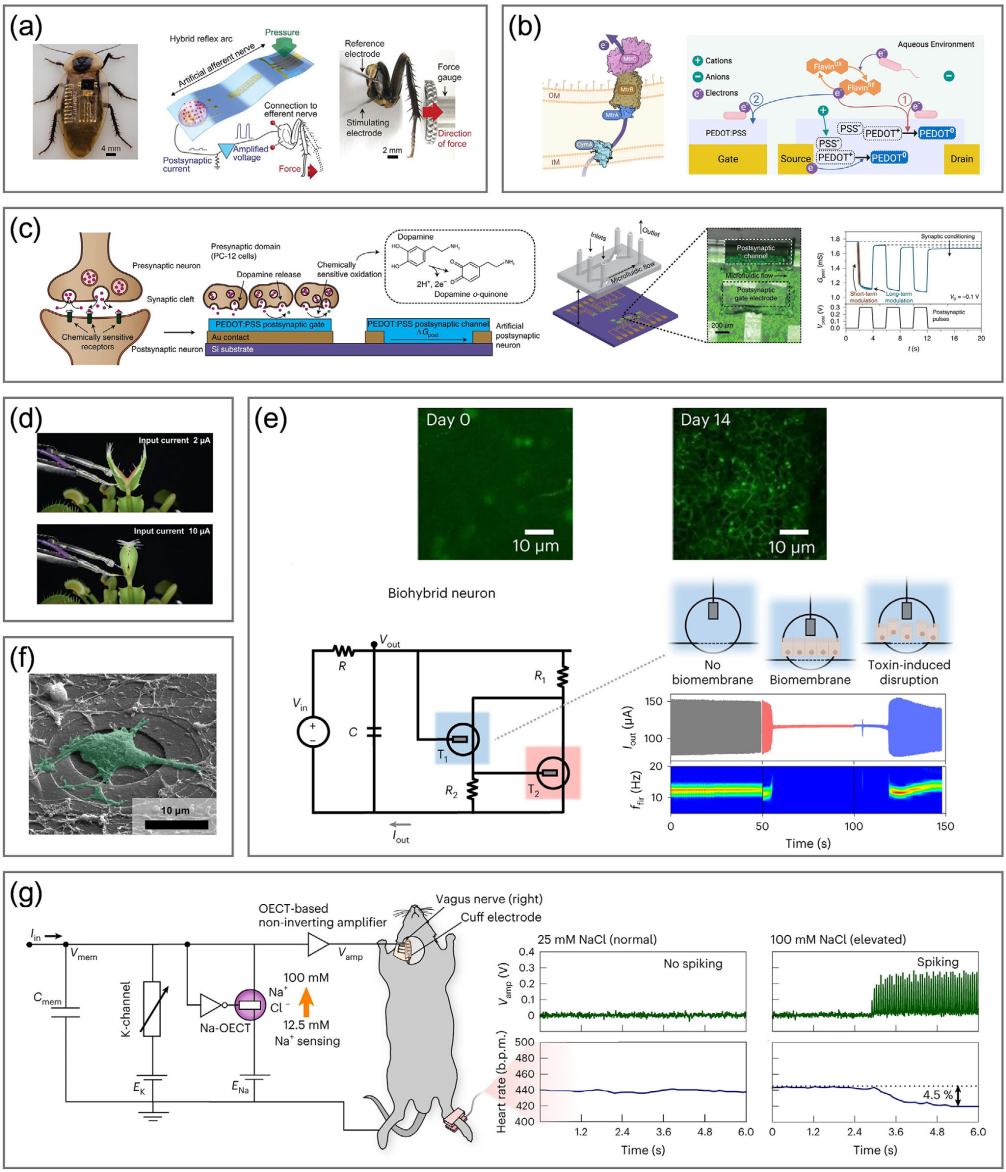
Biointerfaces based on OECSs and OECNs. a) Biointerfaces for movement control by connecting artificial afferent nerves to the leg of a cockroach. Reproduced with permission.^[^ ^13^ ^]^ Copyright 2018, American Association for the Advancement of Science. b) Biointerfaces for electroactive bacterium detection based on the EET of MR‐1 bacteria. Reproduced with permission.^[^ ^163^ ^]^ Copyright 2024, Springer Nature. c) Biointerfaces for dopamine detection by connecting PC‐12 cells to the gates of OECSs. Reproduced with permission.^[^ ^75^ ^]^ Copyright 2020, Springer Nature. d) Biointerfaces with a venus flytrap by LIF‐OECNs. Reproduced with permission.^[^ ^80^ ^]^ Copyright 2022, Springer Nature. e) Biointerfaces for biofilm integrity detection of Caco‐2 cells by OEND‐OECNs. Reproduced with permission.^[^ ^156^ ^]^ Copyright 2022, Springer Nature. f) Rat primary cortical neurons growing on the surface of 1T‐OECNs and forming synaptic networks. Reproduced with permission.^[^ ^72^ ^]^ Copyright 2025, Springer Nature. g). Biointerfaces for sodium ion concentration and heart rate detection with the vagus nerve of a mouse by C‐OECNs. Reproduced with permission.^[^ ^198^ ^]^ Copyright 2023, Springer Nature.

A hybrid monosynaptic reflex arc is constructed by connecting an artificial afferent nerve to the biological efferent nerve of a cockroach (**Figure** **12**a).^[^ ^13^ ^]^ Upon detecting the pressure stimulus, the artificial afferent nerve triggers signal processing in the neuromorphic circuit, which subsequently transmits a biomimetic postsynaptic oscillating signal to the tibial extensor muscle of a cockroach in the leg. The number of activated muscle fibers and the force generated by each muscle fiber increase with the stimulus signal amplitude and frequency, respectively, indicating that the system may be used to control biological muscle movements. These results suggest potential applications in neural prosthetics and biomedical engineering, particularly in the development of advanced prosthetic control systems capable of responding to natural nerve signals.

Recently, a biohybrid synapse formed by a PEDOT:PSS‐based OECS exhibiting dopamine‐mediated plasticity was reported by Keene et al (Figure 12c).^[^ ^75^ ^]^ Dopamine secreted by PC‐12 cells serves as the presynaptic stimulus and undergoes local oxidation at the postsynaptic gate electrode, modulating its conductance state and thereby inducing ion flow in the aqueous electrolyte. The altered channel conductance emulates the neurotransmitter modulation of synaptic weight, whereas the dopamine oxidation process emulates postsynaptic receptor binding in biological synapses. Dopamine and its oxidation product can be readily recycled via a polydimethylsiloxane (PDMS) microfluidic channel, replicating the secretion‐recycling pathways of dopamine in biological synapses. The synaptic weight of the biohybrid synapse can be dynamically adjusted in response to the local neurotransmitter activities, enabling both STP and LTP in accordance with the Hebbian learning rule. Another example of cell‐based detection involves the extracellular electron transfer (EET) of electroactive bacteria (Figure 12b).^[^ ^163^ ^]^ MR‐1 bacteria can interact with the gate and channel regions of OECSs via direct and indirect EET mechanisms. The MR‐1‐seeded OECSs exhibit a distinctive hysteresis loop upon cycling of the gate voltage, showing a short‐term increase in synaptic weight in response to positive gate pulses, while displaying a negligible response to negative pulses, which is asymmetric, providing the basis for STP.

The first attempt of OECN‐based biointerfaces comes to implanting a biomimetic OECN device onto a venus flytrap, in which the input current is modulated to produce a high‐frequency OECN output spiking signal to regulate the closure of the venus flytrap and confirm the compatibility of OECNs with biological neural signals (Figure 12d).^[^ ^80^ ^]^ Later, Sarkar et al. proposed embedding a Caco‐2 cell layer between the gate and channel of a transistor in the OEND‐OECN to form an electrolyte–biofilm–OECT interface for barrier integrity detection (Figure 12e).^[^ ^156^ ^]^ The OECN cannot produce a spiking current when the biofilm is intact because the tight connections of Caco‐2 cells block ions in the electrolyte from moving through the biofilm, preventing gate control of the channel current. Upon exposure to hydrogen peroxide, the disruption of tight connections restores ionic permeability, thereby reinstating current spikes. Furthermore, experiments exhibit that rat primary cortical neurons grow normally on the surface of 1T‐OECN and form synaptic networks after 4 days, demonstrating the nontoxicity and biocompatibility of BBL polymers (Figure 12f).^[^ ^72^ ^]^ The device dimensions are compatible with cortical neuron cytosol (10–20 µm) and axon diameters (0.1–2 µm), supporting future subcellular biointerface development. Additionally, by connecting a C‐OECN to the right cervical vagus nerve of a mouse via cuff electrodes, nerve stimulation is triggered in response to sodium ion concentration (e.g., emulating a high‐salt environment in cystic fibrosis) and results in reduced heart rate, demonstrating its potential in closed‐loop physiological regulation (Figure 12g).^[^ ^198^ ^]^ The implementation of these neuromorphic biointerfaces demonstrates promise in facilitating biorealistic, in vitro, and in vivo integration of ANNs and living biological systems.

### 4.6 Flexible Electronics

Most existing OECT‐based neuromorphic devices that rely on aqueous electrolytes struggle to replicate the intrinsic flexibility of biological neural systems. Therefore, research on flexible innovations in materials, device structures, and operating mechanisms is eagerly demanded.^[^ ^66^, ^260^, ^261^, ^262^, ^263^ ^]^

**Figure 13:**
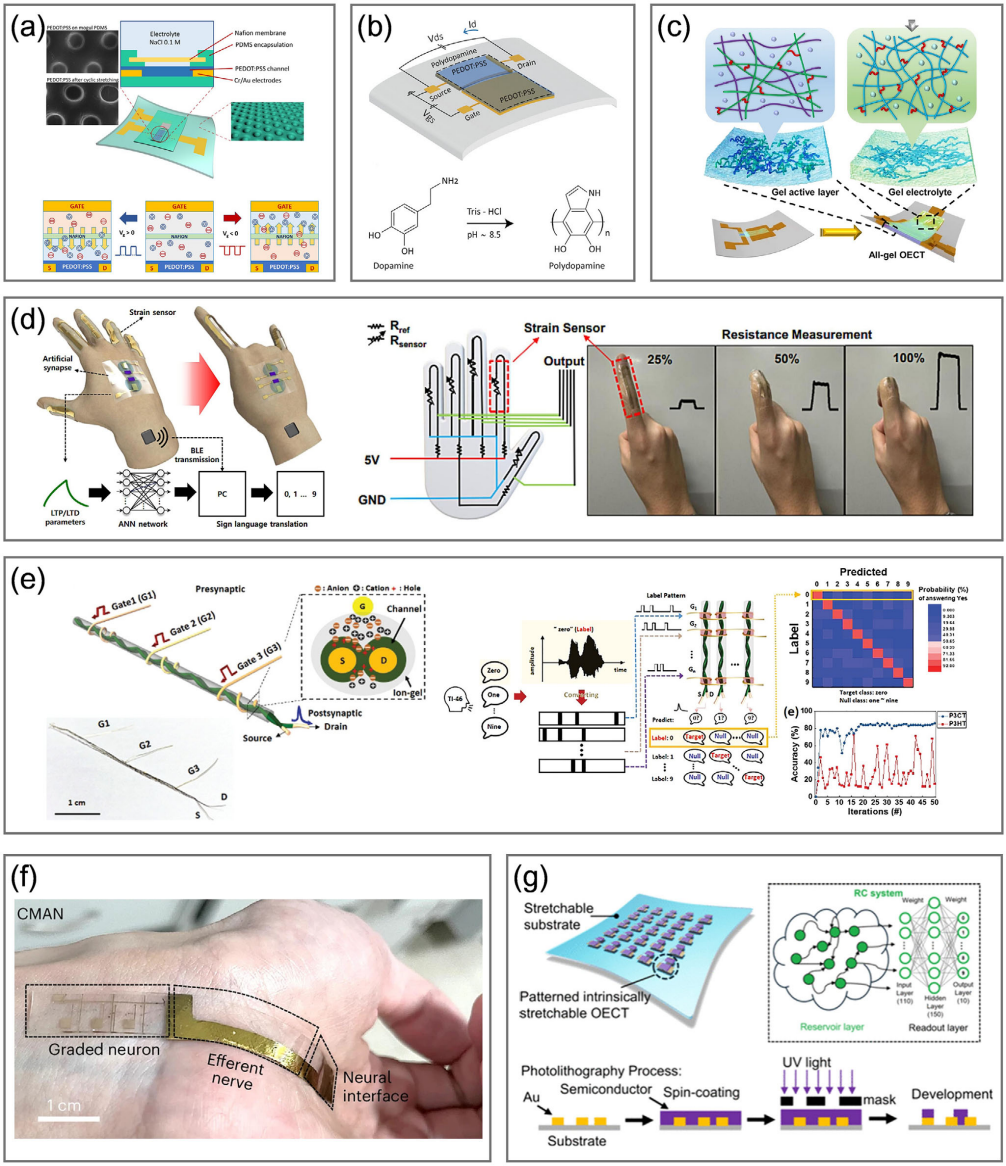
Flexible electronics based on OECSs and OECNs. a) Inserting a solid Nafion membrane between the electrolyte and the channel in flexible OECSs.^[^ ^229^ ^]^ Copyright 2022, Wiley‐VCH. b) Coating polydopamine onto the surface of the channel in flexible OECSs. Reproduced with permission.^[^ ^264^ ^]^ Copyright 2022, Wiley‐VCH. c). Stretchable all‐gel OECSs. Reproduced with permission.^[^ ^265^ ^]^ Copyright 2025, Springer Nature.d) Flexible OECSs in an integrated system for real‐time sensing, training, and reasoning of finger movement. Reproduced with permission.^[^ ^235^ ^]^ Copyright 2024, Elsevier. e) SNNs based on fibrous OECSs and OECNs for speech recognition.^[^ ^226^ ^]^ Copyright 2021, Wiley‐VCH. f) Flexible artificial nerves by integrating an OECN, an OECS, an artificial efferent nerve, and a neural interface. Reproduced with permission.^[^ ^196^ ^]^ Copyright 2025, Springer Nature. g) Scalable fabrication of flexible OECSs with a photo‐cross‐linking strategy into the channel material. Reproduced with permission.^[^ ^238^ ^]^ Copyright 2025, American Chemical Society.

Zhu et al. fabricated a flexible OECS utilizing poly[(bithiophene)‐alternate‐(2,5‐di(2‐octyldodecyl)‐3,6‐di(thienyl)pyrrolyl pyrrolidone)] (DPPT‐TT) as the channel material and PMMA/LiClO_4_ as the solid‐state electrolyte on an ITO/PI substrate, achieving several synaptic functionalities, including excitatory postsynaptic current (EPSC), STP, and LTP.^[^ ^191^ ^]^ The device shows excellent flexibility by maintaining consistent electrical performance even after mechanical bending and conforming to curved surfaces, such as glass bottles. Notably, the device retains its ability to emulate synaptic behaviors under low‐voltage operation in the bent condition, demonstrating its robustness in flexible environments. By incorporating (3‐glycidyloxypropyl)trimethoxysilane (GOPS) cross‐linker and xylitol into PEDOT:PSS for enhanced mechanical stability and utilizing PDMS substrate to effectively absorb mechanical stress, Nguyen et al. demonstrated stable and reliable synaptic functionalities under the stretching condition (**Figure** **13**a).^[^ ^229^ ^]^ Besides, inserting a solid Nafion membrane between the electrolyte and the channel significantly extends the retention time of ions in the channel, enhancing the synaptic weight and stability. Another method to employ PEDOT:PSS‐based OECSs for flexible electronics involves coating polydopamine onto the surface of the channel (Figure 13b).^[^ ^264^ ^]^ Beyond serving as the solid electrolyte, polydopamine partially penetrates into the PEDOT:PSS channel, de‐doping and stabilizing the channel conductance state. With stretchable poly(styrene‐block‐isobutylene‐block‐styrene) (SIBS) as the substrate, this flexible and all‐solid‐state device configuration exhibits great potential for future in vivo applications and large‐scale array integration. In another method, Lu et al proposed a stretchable all‐gel OECS utilizing a double‐network semiconducting polymer gel consisting of PEDOT:PSS and poly acrylamide (PAM) as the channel material and poly(ionic liquid) (PIL) ionogel as the electrolyte (Figure 13c).^[^ ^265^ ^]^ In addition to the ionic liquid improving the channel morphology and increasing the efficiency of charge transport, the all‐gel network expands the interfacial area and ion transport routes.

Flexible OECSs have been shown to function for real‐time sensing in integrated systems. Yoon et al. fabricated an intrinsically stretchable OECS based on P3HT/styrene‐ethylene‐butylene‐styrene (SEBS) as the channel and PVDF‐HFP/EMIM:TFSI as the solid electrolyte (Figure 13d).^[^ ^235^ ^]^ The strain sensor captures the resistance changes caused by finger movement in real time based on the principle of crack propagation and transmits the data to the ANN composed of an OECS array via Bluetooth connection for training and reasoning, thus realizing wireless translation from sign language to text.

To improve the performance of flexible devices and further emulate the structure of neural networks, Kim et al. fabricated a fibrous OECT based on poly[3‐(6‐carboxyhexyl)thiophene‐2,5‐diyl] (P3CT), concurrently realizing synaptic and neuronal behaviors (Figure 13e).^[^ ^226^ ^]^ The device achieves synaptic connections through physical contact of gate microfibers, resembling the dendritic architectures of biological neurons. In addition to the synaptic behaviors arising from the hysteresis effect, the LIF characteristics are true by tuning the temporal spacing of input gate voltage pulses. Finally, a 10 × 10 fibrous SNN is constructed to enable speech recognition tasks based on the OECTs’ spatiotemporal signal processing capability.

By integrating an OECN, an OECS, an artificial efferent nerve, and a neural interface on the flexible PDMS substrate, Wang et al developed a chemically modulated artificial nerve capable of operating under mechanical deformation (Figure 13f).^[^ ^196^ ^]^ The artificial nerve adheres closely to human skin and is well‐suited for minimally invasive implantation, either on the body surface or internally. Despite being fabricated on a flexible substrate, the artificial nerve demonstrates performance comparable to the rigid counterparts, with a nonvolatile memory frequency of 100 kHz and a volatile response time of 27 µs.

A pivotal barrier to the advancement of flexible neuromorphic electronics remains the scalable fabrication of OECTs, confining their development to multi‐component sensing and massive data processing. Recently, Sun reported a successful implementation of scalable fabrication of intrinsically stretchable OECSs (Figure 13g).^[^ ^238^ ^]^ Introducing the photo‐cross‐linking strategy into the channel material p(g2T‐T) enables high‐resolution patterning and stretchability without sacrificing electrical performance induced by cracking. The resulting channel films withstand up to 200% strain while maintaining high conductivity and mechanical integrity, resolving the contradiction between patterning and stretchability of traditional fabrication methods. The array achieves an accuracy of 90.81% and 90.65% in MNIST image classification under 0% and 100% strain, respectively. Moreover, high‐density OECS arrays (>1000 devices cm^−^^2^) are successfully fabricated with a yield of 98% and low device‐to‐device and batch‐to‐batch variability, demonstrating a scalable route toward large‐area and flexible neuromorphic systems.

## 5 Conclusions and Outlook

In conclusion, OECNs, OECSs, and their integrated devices have witnessed tremendous advancements over the past decade, enabling their extensive applications in diverse fields, including neuromorphic computing and flexible biointerfaces. OECNs, based on the four categories of neuron models, have demonstrated the capability for event‐based signal perception, processing, and transmission. Benefiting from the ionic–electronic coupling inherent to OMIECs, OECNs are particularly adept at biochemical sensing and can be tailored for specific functions, including excitation, sensing, and actuation. OECSs have revealed great potential in memory and learning, with state retention and STDP forming the fundamental mechanisms, respectively. Massive studies have aimed to enhance state retention to achieve LTP through material and structural innovations, while STDP has been correlated with Pavlovian conditioning to explore the feasibility of associative learning in hardware‐based electronic systems. Moreover, integrated devices and arrays comprising OECNs and OECSs have constructed hardware‐level ANNs, facilitating applications such as pattern recognition, biointerface construction, and flexible electronics, thanks to their multimodal sensing capability, excellent biocompatibility, and flexibility.

Nevertheless, several critical challenges remain to be addressed. The first challenge concerns the performance of OECTs. Standing at the material level, owing to the ion invasion, excessive swelling, and oxidation in aqueous electrolytes, OMIECs suffer from inadequate stability, imposing restrictions on their long‐term applications for sensing in OECNs and memory in OECSs. Although issues related to swelling and oxidation in OMIECs might be slightly relieved through the employment of solid electrolyte,^[^ ^266^, ^267^ ^]^ backbone and side chain engineering,^[^ ^124^, ^154^, ^268^ ^]^ and ladder polymers,^[^ ^153^, ^155^, ^195^ ^]^ further efforts are demanded for improving the stability of OMIEC materials effectively and readily. Despite the fact that n‐type OMIECs have witnessed dramatic advancements in recent years,^[^ ^102^, ^153^, ^196^, ^268^ ^]^ their steady‐state and transient performance still falls behind their p‐type counterparts, significantly limiting the development of complementary circuits and neuromorphic devices. Additionally, to address biocompatibility concerns that potentially trigger inflammatory responses upon implantation due to the stiffness incongruence between OMIECs and biological tissues, tailored and customized material engineering innovations are expected to mitigate inflammatory responses, paving the way for further realistic biointerface applications.^[^ ^196^ ^]^ Standing at the device level, on account of the slow dynamics of ion migration between the OMIEC channel and electrolyte and the capacitance effect induced by the accumulation of ions at the interface,^[^ ^269^ ^]^ OECTs generally suffer from slow response speed, which can be improved through enhancing the ionic mobility and reducing the channel length of the OMIEC channel. Furthermore, downsizing is essential to reduce the parasitic capacitance in OECTs to enhance the firing frequency of OECNs and minimize the response time of OECSs to better match biological dynamics. However, in terms of fabrication techniques, traditional photolithography lacks the adaptability necessary for developing sub‐micron‐scale devices. Therefore, combining advanced manufacturing technologies, such as electron‐beam lithography^[^ ^247^ ^]^ and 3D printing^[^ ^270^, ^271^ ^]^ to achieve efficient and low‐cost fabrication of sub‐micron‐scale devices with high performance is highly desirable. Vertical architectures offer an alternative method to reduce the channel length to the nanoscale, significantly improving both the steady‐state and transient performance,^[^ ^101^, ^102^ ^]^ yet the fabrication complexity and emerging short‐channel effects remain to be resolved. Another critical problem is related to the solid‐state integration that is indispensable in wearable electronics and high‐density systems, consisting of interfacial defects and electrode degradation. Developing interface engineering, buffer layer, and efficient encapsulation are eagerly demanded to resolve the problem.

The second challenge is regarding the functionalities of OECNs and OECSs. Given the intricate complexity of the human neural system, the four realized OECN models are not capable of completely replicating the dynamic features of biological neurons, such as spike frequency adaptation and inhibition‐induced spiking.^[^ ^73^ ^]^ Hence, improving existing OECN models with extra ion channels and introducing OECTs into other biorealistic neuron models, such as Morris–Lecar and Izhikevich models, indicates a promising direction of future development. It is challenging to trade off the functionality, complexity, and power consumption when optimizing OECN models. Considering that all OECN models consist of several OECTs, the instability of every OECT in practical trials and the performance mismatch between p‐ and n‐type OECTs restrict the feasibility and reliability of sensing and signal transmission. Besides, biological neurons feature processing multiple stimuli simultaneously, such as biochemical, vision, and touch; however, existing OECNs are mostly designed for single stimuli and lack multimodal perception and integrated processing capability.^[^ ^156^, ^157^, ^198^ ^]^ Likewise, existing OECSs generally lack synergistic regulation by multiple neurotransmitters found in biological synapses.^[^ ^75^, ^159^ ^]^ Moreover, there is an intrinsic contradiction between sensing (volatile) and memory (nonvolatile) in OECSs, as sensing requires a low ion transport barrier for fast response, whereas memory requires a high barrier to maintain the conductance state. Although several approaches have proven effective in decoupling the two process,^[^ ^164^, ^233^ ^]^ the complexity and precision of the modulation hinder their widespread applicability. Therefore, exploring novel and feasible means for dynamic and bidirectional switching of the two modes is urgently required. Additionally, biological synapses universally exhibit quasi‐continuously adjustable weights, while the weights of OECSs present nonlinearity and discretization induced by the incapability of precise control of ionic migration. Furthermore, the lack of comprehensive theoretical frameworks for OECT dynamics restricts the in‐depth physical correlation between OECSs and biological synapses, and the physical models for different types of materials and devices exhibit significant variations. Hence, constructing reliable physical models that meticulously describe the electronic and ionic dynamics not only facilitates quantitative regulation of electronic and ionic behaviours but also helps to establish further biorealistic OECSs and their simulation platforms.

The third challenge pertains to the construction and operation of integrated systems. Large‐scale integration of OECT‐based neuromorphic electronics remains obstructed by the device‐to‐device variability. Optimized manufacturing methods, such as photo‐cross‐linking strategy^[^ ^238^, ^272^ ^]^ and directional growth with AC‐electropolymerization,^[^ ^273^ ^]^ may effectively improve the uniformity of devices across a large scale, offering scalable manufacturing pathways. Another critical challenge lies in the difficulty of reproducing the 3D folding structures of biological neural networks, which are closely associated with their complicated functionalities. Furthermore, potential issues concerning the interconnection, signal synchronization, and energy supply also need to be settled in high‐density systems. Moreover, existing neural networks based on whether OECTs or OFETs are at small scales compared to their CMOS counterparts and the computing process is operated on peripheral software, where miniaturized and biorealistic in‐sensor computing integration is still under innovative potential. Since the development of OECT‐based neuromorphic systems is still in its early stages, drawing inspiration from the mature technologies that have gained decades of evolvement, such as CMOS and memristors, might offer potential solutions to address relevant issues in OECT‐based counterparts. As for future development, the hybrid integration of OECT‐based neuromorphic devices with biological tissues or mature CMOS‐based neuromorphic devices opens prospective opportunities for applications in biointerfaces and computational systems by combining their respective advantages. With continued interdisciplinary research and advancements, OECT‐based neuromorphic devices might revolutionize the fields of bioelectronics as well as neuroscience in the near future.

## Conflict of Interest

The authors declare no conflict of interest.
